# Experimental and computational analyses for elucidation of structural, electronic, thermal, and vibrational properties of ethionamide crystal

**DOI:** 10.1038/s41598-025-29051-w

**Published:** 2026-01-04

**Authors:** Raychimam D. S. Bezerra, Ketelly E.da S. Alves, Jailton R. Viana, Luzeli M. da Silva, Mateus R. Lage, Rossano Lang, Adenilson O. dos Santos, Eliana B. Souto, João G. de Oliveira Neto

**Affiliations:** 1 Center for Social Sciences, Health and Technology, Federal University of Maranhão-UFMA, Imperatriz, MA 65900-410 Brazil; 2 Institute of Science and Technology, Federal University of São Paulo-UNIFESP, São José Dos Campos, SP 12231-280 Brazil; 3 UCD School of Chemical and Bioprocess Engineering, University College Dublin, Belfield, Dublin 4, D04 V1W8 Ireland; 4 Center for Science of Imperatriz, Federal University of Maranhão-UFMA, Imperatriz, MA 65900-410 Brazil

**Keywords:** Ethionamide, DFT calculations, Hirshfeld surface, Vibrational spectroscopy, Drug optimization, Chemistry, Computational science

## Abstract

Ethionamide (ETH) is a second-line drug widely used to treat multidrug-resistant tuberculosis (MDR-TB), but its low aqueous solubility compromises bioavailability and limits its therapeutic efficacy. To understand the ways to improve these properties, we combined experimental and computational approaches to elucidate the structural, electronic, thermal, and vibrational properties of ETH crystals. Powder X-ray diffraction analysis revealed a monoclinic crystal system (C1*c*1-space group), stabilized by intermolecular interactions, primarily H⋯H (49.3%) and H⋯S/S⋯H (22.1%) contacts. Energy framework analysis revealed the anisotropic nature of intermolecular interactions, with dispersion forces accounting for approximately 60% of the total stabilization energy, while Coulombic interactions showed significant directionality along the crystallographic *a*-axis and within the *bc*-plane. The total energy framework indicated that the strongest stabilization propagates along the *b*-axis, suggesting the formation of highly stable molecular chains, which directly influence crystal morphology and dissolution behavior. Thermal analysis confirmed ETH stability up to 162.2 °C, with melting and decomposition events characterized by endothermic peaks. Density functional theory (DFT) calculations confirmed ETH high electronic gap (7.84–8.09 eV), indicating low reactivity, while solvation studies highlighted its greater stability in polar solvents like water and methanol. Theoretical nuclear magnetic resonance studies (^1^H and^13^C) showed minimal solvent influence on chemical shifts, reinforcing the structural stability of ETH across environments. Vibrational spectroscopy, supported by DFT, identified key modes associated with the pyridine ring, NH_2_, and C=S groups. Hirshfeld surface analysis further revealed the dominance of hydrogen bonds and van der Waals interactions, with minimal void space (5.3%) in the crystal lattice. Electrostatic potential maps identified electron-rich regions around nitrogen atoms as potential sites for hydrogen bonding and protonation, which are relevant for pharmacological interactions. These findings offer critical insights for optimizing ETH’s solid-state properties to enhance its solubility and bioavailability, paving the way for improved formulations against MDR-TB.

**Supplementary Information:**

The online version contains supplementary material available at 10.1038/s41598-025-29051-w.

## Introduction

Tuberculosis (TB) continues to be the second most prevalent infectious disease after COVID-19, resulting in substantial mortality on a global scale. According to the 2023 Global TB Report by the World Health Organization (WHO), in 2022, an estimated 10.6 million people fell ill with TB^1^. Isoniazid, ethambutol, rifampicin, and pyrazinamide constitute the primary pharmaceutical agents utilized during the initial phase of treatment for *Mycobacterium tuberculosis* infection^2–5^. These standard frontline medications exhibit limited efficacy in treating multidrug-resistant tuberculosis (MDR-TB)^6,7^. Thioamides, including ethionamide (ETH) and prothionamide, have shown superior effectiveness against MDR-TB, with ETH that bears a structural resemblance to isoniazid (INH), being particularly noteworthy for its enhanced therapeutic properties, cost-effectiveness, and widespread availability as a second-line medication^8–10^.

ETH, also known as 2-ethyl-4-thioamidopyridine or 2-ethylthioisonicotinamide, is a chemical compound with a pyridine ring, an ethyl–methyl chain, and a thioamide^11^. It is classified as a Class-II drug under the Biopharmaceutical Classification System (BCS) due to its low aqueous solubility and high permeability^12^. ETH is also used in a combined chemotherapy protocol for leprosy^13^. However, its limited solubility in water and bioavailability have led to the development of novel solid forms to enhance its pharmaceutical efficacy^14–17^. This development process is complex and resource-intensive, requiring a deep understanding of the physicochemical properties of the drug candidate. Here, computational methodologies play a crucial role, helping to disclose the characteristics of this pharmaceutical agent and providing precise insights into its practical applications^18^.

Electronic structure calculations have become common in the scientific community for analyzing chemical systems to investigate their structural and thermodynamic properties^19^. Density functional theory (DFT) is one of the most prevalent methods in exploring physicochemical and electronic properties^20–22^. Specifically, DFT has proven valuable in research endeavors involving the determination of crucial descriptors for chemical reactivity, such as ionization potential (*IP*), electron affinity (*EA*), electronegativity (*χ*), and other properties in molecular structures^23,24^.

Additionally, one of the computational methods extensively employed to study the intermolecular interactions within crystalline materials is the Hirshfeld surface analysis^25,26^. Through a detailed examination of atom-to-atom contacts, the Hirshfeld surface analysis offers a comprehensive insight into the arrangement of molecules within a crystalline lattice^27^. The Hirshfeld surface is generated as a function of the total electron density of individual atoms within a given species, divided by the cumulative electron density of their closest neighboring atoms^28–30^. This projection is constructed based on a weight function *w*(*r*), which compares the electron density contribution of a molecule (the promolecule, formed by the sum of non-interacting atoms) to the total electron density of the crystal (the procrystal)^31^.

From this context, in this present investigation, the structural, thermal, and vibrational properties of ETH crystals produced via slow solvent evaporation were examined using powder X-ray diffraction (PXRD), thermogravimetry (TG), differential thermal analysis (DTA), differential scanning calorimetry (DSC), Fourier-transform infrared (FT-IR), and Raman spectroscopy. Computational approaches relying on DFT and Hirshfeld surface analysis were utilized. Experimental vibrational data were compared to the theoretical estimations of the ETH molecule in various solvent environments.

## Experimental and theoretical procedures

### Crystals growth

ETH crystals were synthesized using the slow solvent evaporation method, as previously described by us^14^. Briefly, a precursor solution containing 0.1 mol/L of ETH (Sigma-Aldrich, ≥ 98%, Darmstadt, Germany) in 30 mL of methanol (Sigma-Aldrich, 99.8%, St. Louis, MO, USA) was prepared under constant magnetic stirring at 360 RPM. After complete solubilization, the solution was filtered through a 25 μm filter paper (Sigma-Aldrich, St. Louis, MO, USA) and transferred to a container covered with plastic film perforated with 25 randomly distributed holes, allowing controlled solvent evaporation and nucleation of the solid phase. After 5 days, orange-colored prismatic crystals were successfully obtained. Furthermore, the crystallization process was reproducible across multiple batches, with crystal formation typically occurring within 5 ± 1 days under controlled conditions (35.9 ºC, 45 ± 5% relative humidity).

### Characterization techniques

The crystal structure of ETH was investigated using PXRD with a PANalytical diffractometer (Empyrean model, Malvern Panalytical, Malvern, UK) operating at 40 kV/40 mA with Cu–K_α₁_ radiation (λ = 1.5418 Å). The diffractogram was collected in the angular range of 5–45° (2θ) with a step size of 0.02° and a counting time of 2 s/step. Subsequently, the PXRD pattern was analyzed using the Rietveld refinement method in the GSAS software^32^, employing lattice parameters previously reported in the literature^33^. For that, the unit cell parameters, atomic positions, isotropic thermal factors, and profile parameters were refined. No bond length or angle constraints were applied, as the initial structure (1150421) was already in good agreement with the experimental data.

TG-DTA and DSC experiments were conducted using, respectively, DTG-60 and DSC-60 thermal analyzers (Shimadzu, Kyoto, Japan). For TG-DTA measurements, the powdered crystals were evenly distributed in a platinum crucible and heated at 5 °C/min in the range of 25–300 °C (± 0.5 °C) under a nitrogen atmosphere (100 mL/min). For DSC analysis, the powdered sample was placed in an alumina crucible and analyzed at 25–200 ± 1.0 °C (5 °C/min) under a nitrogen flow of 100 mL/min.

Raman spectra were recorded in the wavelength region of 220–3200 cm^− 1^ using a Trivista 557 spectrometer (Princeton Instruments, MO, USA) equipped with a charge-coupled device (CCD) detector. A green solid-state laser (λ = 532 nm) was used as the excitation source on the powdered sample. The Raman spectra were collected with 4 accumulations of 60 s each and a spectral resolution of 2 cm^− 1^.

FT-IR spectra were measured using the KBr (Sigma-Aldrich, 99.8%, St. Louis, MO, USA) pellet method (2%) with a Bruker spectrophotometer (Vertex 70 V model, Karlsruhe, Germany). The FT-IR spectrum was collected in the wavelength range of 4000–400 cm^− 1^, with 32 scans and a spectral resolution of 4 cm^− 1^.

### Theoretical calculations

The initial structural data of ETH was retrieved from the Cambridge Crystallographic Data Centre (CCDC), with reference code 1150421. The refined crystallographic structure was the basis for generating input files for subsequent computational analyses.

To investigate non-covalent interactions, Hirshfeld surface analysis, framework energy, and 2D fingerprint plots were employed using CrystalExplorer software (version 21.5)^34^. Through a detailed examination of atom-to-atom contacts, the Hirshfeld surface analysis offers a comprehensive insight into the arrangement of molecules within a crystalline lattice. The Hirshfeld surface is generated from electron density calculations. It utilizes a weight function that reflects the electron density contribution of a specific molecule in a crystal relative to the total electron density of the surrounding crystal structure^31^. This weight function, often denoted as w(*r*), represents the ratio of the electron density of the promolecule (the molecule of interest, considered as a sum of isolated atoms) to the electron density of the procrystal (the sum of all atomic densities in the crystal)^35^. The function is defined as

$$\:w\left(r\right)=\frac{{\rho\:}_{promolecule}\left(r\right)}{{\rho\:}_{procrystal}\left(r\right)}$$

where *ρ*_promolecule_ (*r*) is the electron density of the promolecule and *ρ*_procrystal_(*r*) is the electron density of the procrystal^34^. The Hirshfeld surface corresponds to the isosurface where w(*r*) = 0.5, demarcating the region where the electron density contribution from the molecule equals that of the surrounding crystal environment. In other words, the Hirshfeld surface encompasses the region where the electron density contribution from the molecule equals (or exceeds) that from the rest of the crystal^34^. From this surface, 2D fingerprint plots can be generated, which display the distribution of contact distances *d*_e_ and *d*_i_ and provide a quantitative breakdown of interaction types, enabling the identification of dominant interaction motifs in the crystal structure. Furthermore, the crystal voids were calculated using electron density isosurfaces with a threshold value of 0.002 a.u. within the primitive unit cell^36^. This analysis allowed for the quantification of empty spaces in the crystal structure, providing insight into potential solvent-accessible regions or structural packing efficiency. The energy frameworks were calculated in the primitive unit cell using a 1 × 2 × 2 supercell configuration and the DFT/6-311 method.

The electronic and structural properties of ETH were investigated using DFT calculations performed with Gaussian 16^37^. The initial molecular geometry was obtained from the experimental crystal structure and subsequently optimized as an isolated monomer. The ωB97X-D functional^38,39^ was selected as it has been specifically benchmarked for organic pharmaceutical compounds and provides accurate geometric parameters, vibrational frequencies, and electronic properties for medium-sized drug molecules^40^. This functional incorporates empirical dispersion corrections, which are crucial for properly describing non-covalent interactions relevant to crystal packing and solvation effects. The ωB97X-D was combined with the 6-311 + + G(d, p), which offers an optimal balance between computational efficiency and accuracy for medium-sized drug molecules like ETH^14,41^. This basis set provides: (*i*) triple-ζ quality for valence electrons (6-311), (*ii*) diffuse functions (++) to properly describe electron density distribution, and (*iii*) polarization functions (d, p) to accurately model molecular orbitals and intermolecular interactions. Calculations were carried out in vacuum, water (*ε* = 78.36), methanol (*ε* = 32.63), and chloroform (*ε* = 4.71), with solvation effects modeled using the Integral Equation Formalism Polarizable Continuum Model (IEFPCM) implicit solvation approach^42^. The united atom topological model was used for cavity construction with default atomic radii. It is important to note that the IEFPCM model treats the solvent as a homogeneous dielectric continuum, effectively capturing bulk polarization effects but not specific solute-solvent interactions such as hydrogen bonding or localized dispersion forces. While this approach provides valuable insights into solvation trends, conclusions regarding solvent-induced structural or electronic changes should be considered qualitative. Future studies employing explicit solvation models or periodic DFT calculations including solvent layers would be valuable to explore specific interactions at the molecular level. From the optimized structure, we derived: frontier molecular orbitals, chemical reactivity indices, thermodynamic properties, geometric parameters, and vibrational modes. Post-processing and data analysis utilized Chemcraft 1.8^43^ for visualization and Multiwfn 3.8^44^ for advanced quantum chemical property calculations. The calculated IR and Raman vibrational frequencies were scaled by a factor of 0.957^45^ to achieve better agreement between theoretical predictions and experimental observations. Vibrational properties were computed for an isolated ETH monomer with IEFPCM; intermolecular interactions and crystal packing effects are not included in this model. Moreover, the nuclear magnetic resonance (NMR) spectra for both^1^H and^13^C nuclei were obtained through the Gauge-Independent Atomic Orbital (GIAO) approach^46^. When accounting for solvation effects, the isotropic shielding values of the ETH molecules were adjusted based on their chemical shifts (expressed in ppm) relative to tetramethylsilane (TMS), which served as the reference compound. The TMS calculations were performed using the same computational setup applied to the ETH molecule, following the procedure outlined by Guzzo et al.^47^. This comprehensive computational approach enabled detailed characterization of the ETH electronic and structural features.

## Results and discussion

### Structural characterization via PXRD and Rietveld refinement

**Fig. 1:**
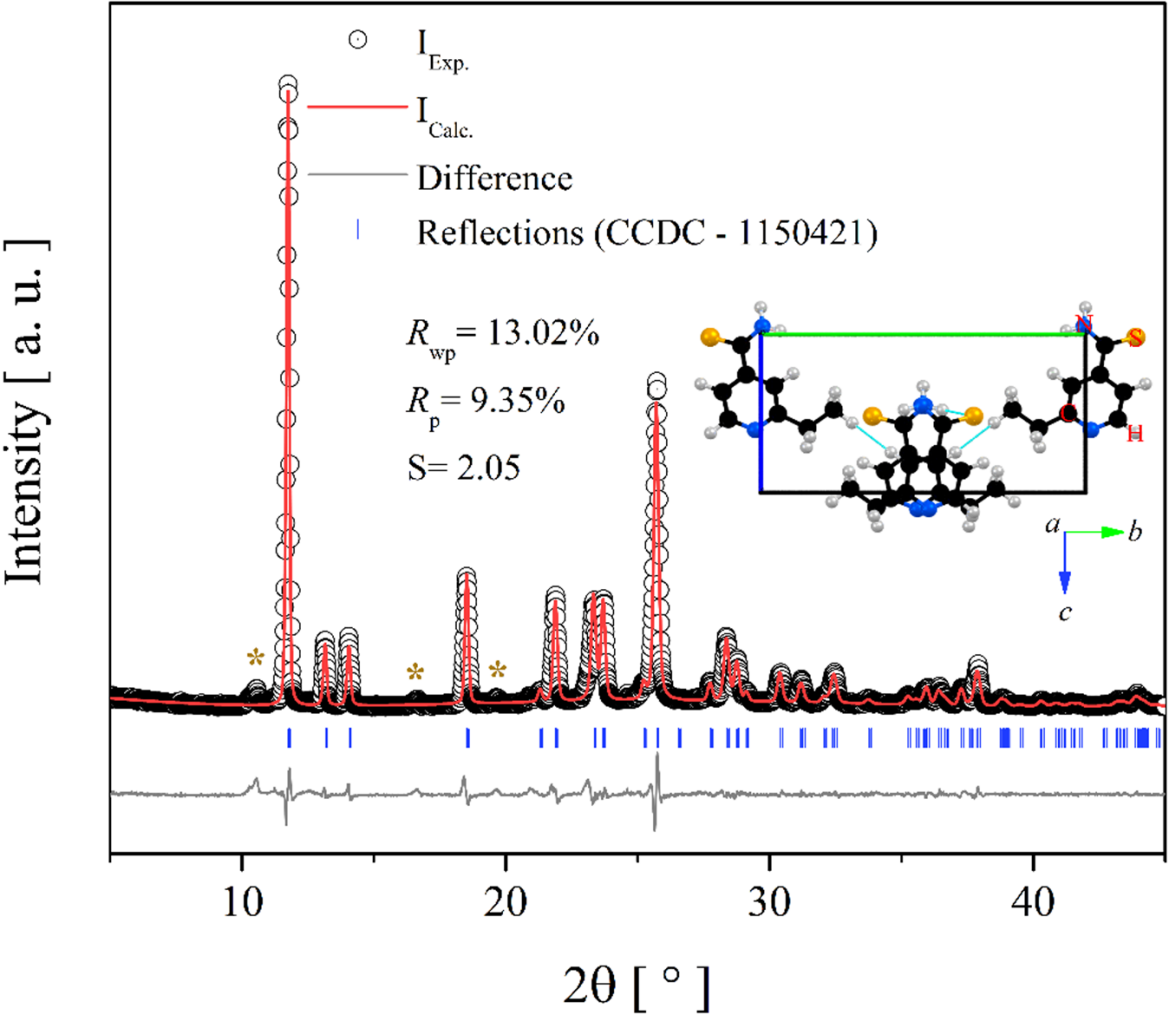
PXRD Rietveld refinement of powdered ETH crystal at room temperature. Brown asterisks represent possible impurities or new polymorphic phases. ***inset***: primitive unit cell of ETH along the *a *and *b* planes showing the intermolecular interactions formed.

Figure 1 shows the PXRD pattern refined by the Rietveld method at room temperature. The refinement quality parameters *R*_wp_ (13.02%), *R*_p_ (9.35%), and *S* (2.05) demonstrate a good agreement with the structural phase previously reported in the literature (*a* = 8.832(2) Å, *b* = 14.996(4) Å, *c* = 7.918(2) Å, *β* = 128.51(5) º, and *V* = 820.605 Å^3^)^33^. The low-intensity residual peaks observed at approximately ≈ 11°, 16°, and 20° can be attributed to minor impurities (< 2% by mass), possibly arising from residual solvent traces or the formation of new polymorphic phases. According to the data obtained, ETH crystallizes in monoclinic symmetry with C1*c*1($\:{C}_{s}^{4}$)-space group in methanol, containing 4 monomers per unit cell (*Z* = 4) and refined lattice parameters: *a* = 7.317(7) Å, *b* = 15.042(5) Å, *c* = 7.931(3) Å, *β* = 109.05(6)º, and *V* = 825.09(8) Å^3^, consistent with the previously reported structure by Alleaume et al.^33^. Although Rajalakshmi et al.^8^ reported a structure in a different setting of temperature, both phases represent the same crystalline form, as confirmed by crystal packing similarity analysis (30/30 molecular match in Mercury)^48^. The minor differences in lattice parameters (e.g., unit cell volume: 825.09(8) Å^3^ in this work vs. 801.01(3) Å^3^ in Rajalakshmi et al.^8^) are likely due to temperature effects (25 °C vs. − 173 °C) and experimental resolution.

Additionally, the *inset* of Fig. 1 illustrates the distribution of ETH monomers within the primitive unit cell. As seen, each organic unit can interact with its surroundings through intermolecular interactions (indicated by light-blue dashed lines) of the C–H∙∙∙H or N–H∙∙∙S type. These contacts are formed between dimers and propagate in the crystal lattice, providing structural stability to the atomic ordering pattern.

### Analysis of Hirshfeld surfaces and their different mappings

**Fig. 2:**
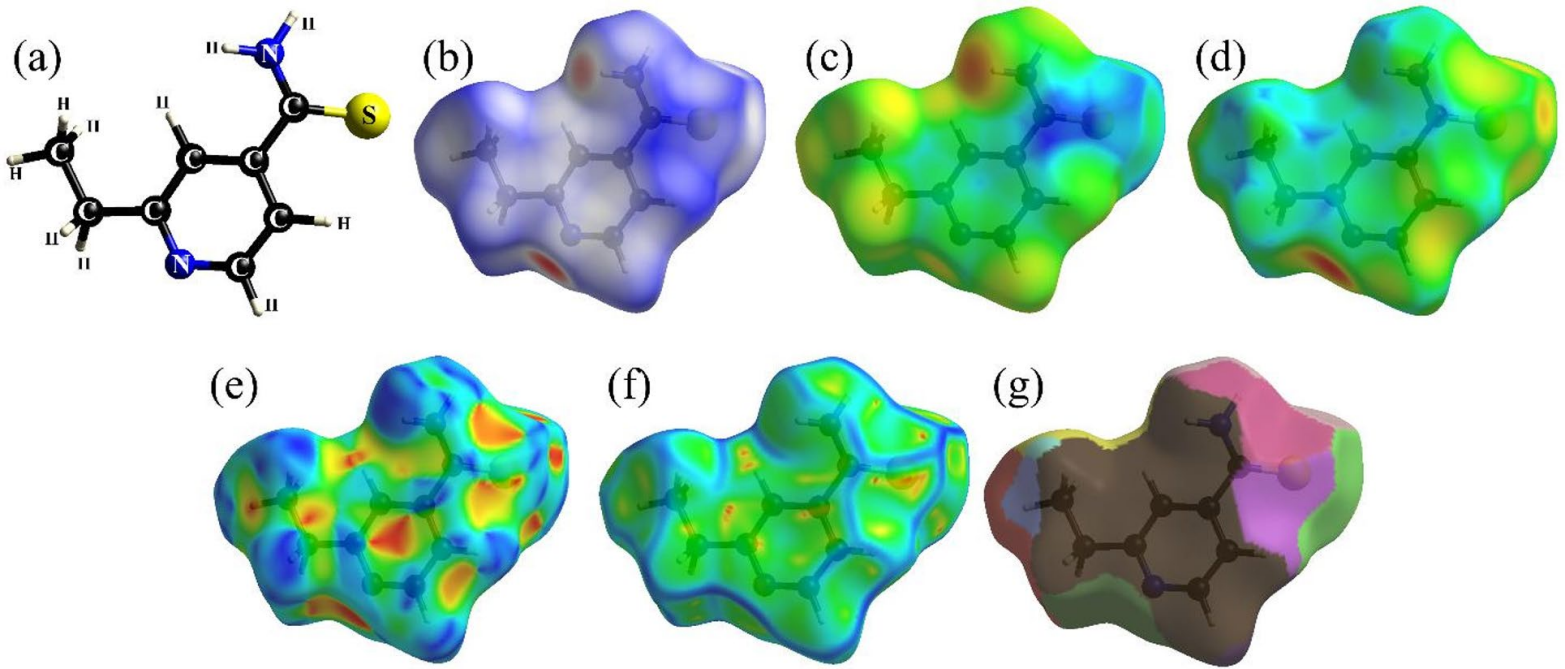
(**a**) EHT molecular unit. Hirshfeld surface of the ETH crystal mapped in terms of (**b**) *d*_norm_, (**c**) *d*_e_, (**d**) *d*_i_, (**e**) shape index, (**f**) curvedness, and (**g**) fragment patch. The plots were generated from CrystalExplorer software (version 21.5).

For a more detailed analysis of the intermolecular interactions between ETH monomers (Fig. 2a) in the crystal lattice, qualitative and quantitative studies were conducted using Hirshfeld surfaces and their different mappings. Figure 2b illustrates the Hirshfeld surface mapped with a color scheme, where the red regions denote close contacts, white regions indicate contacts near the Van der Waals radius, and blue regions represent distant contacts^49^. The reddish areas surrounding the N and H atoms in the aromatic ring and amine group (NH_2_), respectively, highlight sites of strong intermolecular interactions. These are associated with contacts involving hydrogen bonds of the N∙∙∙H/H∙∙∙N type and H∙∙∙H contacts.

The Hirshfeld surfaces plotted in terms of *d*_e_ (Fig. 2c) and *d*_i_ (Fig. 2d) indicate the receiving and donating sites of intermolecular contacts. In this analysis, the surfaces in function of *d*_e_ and *d*_i_ represent, respectively, the shortest distances from the surface to the external nuclei (neighboring molecules) and internal nuclei (the molecule itself). Regions where *d*_e_ is significantly smaller than *d*_i_ indicate sites where neighboring atoms can approach the surface, characterizing acceptor sites of interactions (electronegative atoms, such as N or O). Conversely, regions where *d*_i_ is smaller than *d*_e_ suggest donor sites (hydrogen atoms bonded to N or O).

Additionally, the Hirshfeld surfaces mapped as a function of the shape index, curvedness, and the projected patch properties, as shown in Fig. 2e–g, are linked to structural topological contacts^49,50^. These contacts detail the arrangement of molecular units within the crystal lattice. The reddish triangles depicted in Fig. 2e denote concave regions where the ETH monomers engage in coplanar stacking, predominantly within the alicyclic portion, via shorter and more intense contacts. In contrast, areas shaded in cool color tones characterize convex regions associated with more distant and weaker interactions. The curvedness surface (Fig. 2f) quantifies local morphological features of the Hirshfeld surface, where high-curvedness regions (blue contours) correspond to abrupt curvature (see the ridge-like edges), while low-curvedness areas (green) reflect flatter surfaces. The greenish flat areas correspond to zones of minimal surface curvature, typically associated with extended molecular contacts, such as van der Waals interactions or parallel-displaced π–π stacking in aromatic systems. In ETH, these regions align with the planar pyridine rings, suggesting their role in stabilizing the crystal lattice through dispersive forces. Complementing this, the fragment patch locations shown in Fig. 2g pinpoint areas on the surface where neighboring units overlap to form the propagation of the entire crystal lattice. In summary, the brown areas around much of the ETH interact with another unit in this same region. Combined, these descriptors provide a multidimensional view of packing motifs in ETH crystals.

**Fig. 3:**
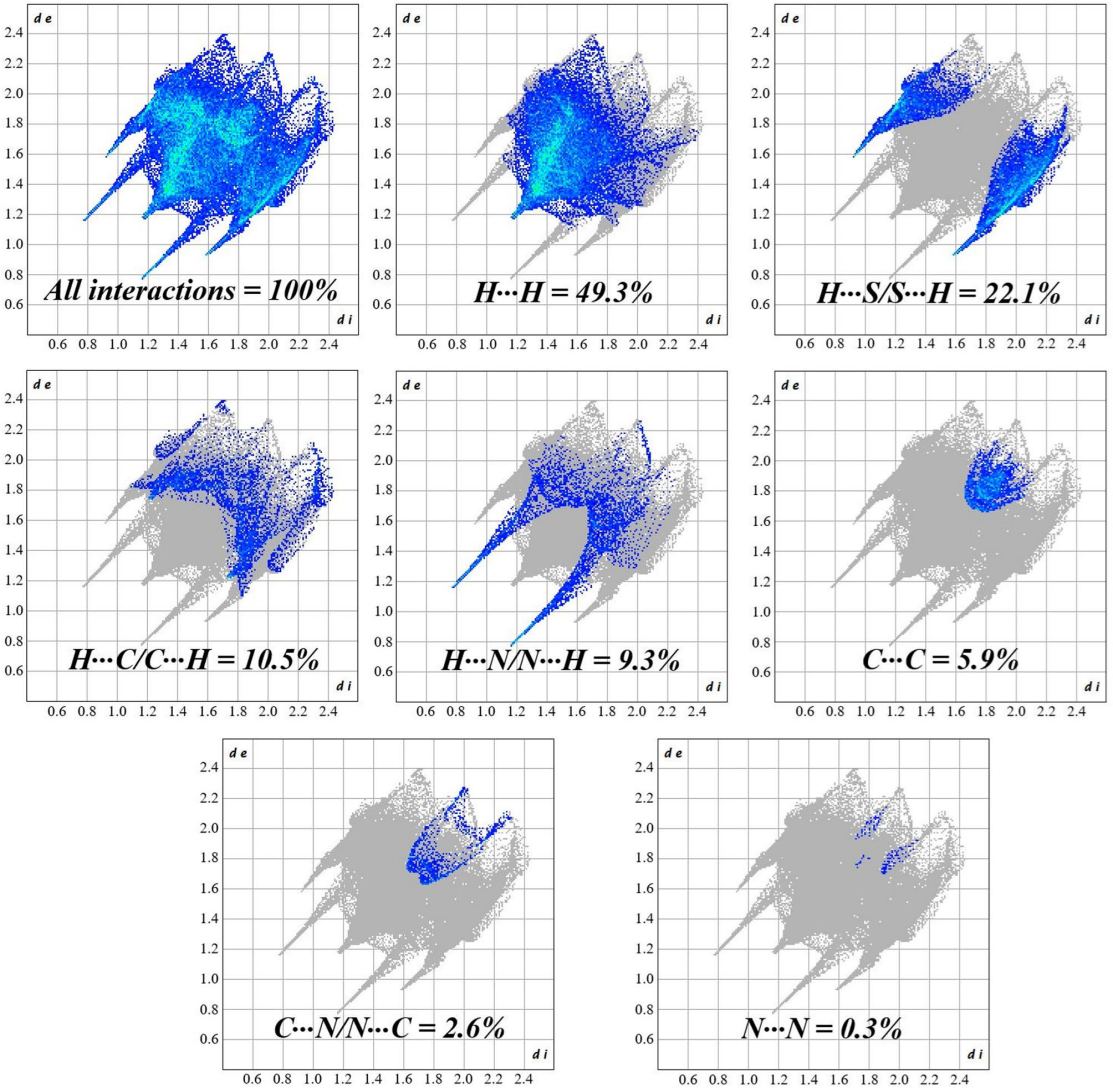
Full and interaction-specific 2D-fingerprint plots of ETH crystal. The plots were generated from CrystalExplorer software (version 21.5).

In addition to the qualitative study on intermolecular contacts, a quantitative analysis was conducted from the total and specific 2D fingerprint plots shown in Fig. 3. The 2D fingerprint decomposition plots display the distribution of surface points as functions of *d*_e_ and *d*_i_ distances. The color gradient (light-to-dark blue) reflects the relative frequency of specific *d*_e_/*d*_i_ pairs, where darker regions correspond to more prevalent contact distances.

The full 2D fingerprint plot is presented in Fig. 3 and specific interaction graphs were generated from it. As shown, the dominant H⋯H (49.3%) and H⋯S/S⋯H (22.1%) contacts represent the most prevalent intermolecular interactions in the crystal structure. Their geometric prevalence suggests potential stabilization of the packing motif. In addition to these, it was verified that the contacts H⋯C/C⋯H (10.5%), H⋯N/N⋯H (9.3%), C⋯C (5.9%), C⋯N/N⋯C (2.6%), and N⋯N (0.3%) contribute relatively on the surface. Sharp, well-defined features at low *d*_e_/*d*_i_ values in the N⋯H/H⋯N plot are characteristic of directional hydrogen bonds.

The calculated data on intermolecular interactions here, obtained through descriptive analysis of the Hirshfeld surface, are consistent with the results reported by Rajalakshmi et al.^8^, showing only minor discrepancies in the percentage contributions of contacts. Notably: (*i*) a slightly higher contribution from H⋯S/S⋯H contacts (+ 2.1%), suggesting stronger interactions involving the thioamide group; and (*ii*) a reduced occurrence of H⋯N/N⋯H contacts (-10%), reflecting variations in the hydrogen bonding lattice. Possibly, these subtle differences may be attributed to the temperature at which the structural determination was performed.

**Fig. 4:**
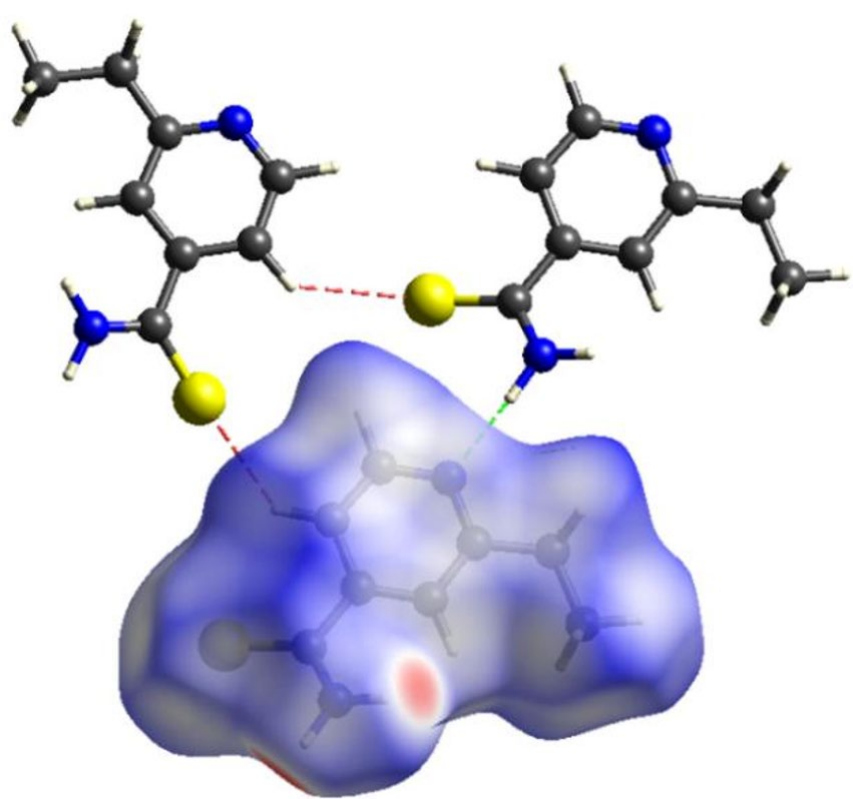
Specific contacts of ETH crystal: red (H⋯S/S⋯H – 2.984 Å) and green (H⋯C/C⋯H – 3.204 Å) lines. The plot was generated from CrystalExplorer software (version 21.5).

Figure 4 illustrates a detailed model of how ETH monomers interact with each other based on specific contacts. The red and green dashed lines characterize the intermolecular interactions H⋯S/S⋯H and H⋯C/C⋯H, respectively. The H⋯S/S⋯H (2.984 Å) and H⋯C/C⋯H (3.204 Å) contacts identified in surface fall within the expected range for intermolecular interactions in organic crystals. These values are consistent with previous studies on ETH compounds, supporting the stability of the crystal structure^8,11,51^.

**Fig. 5:**
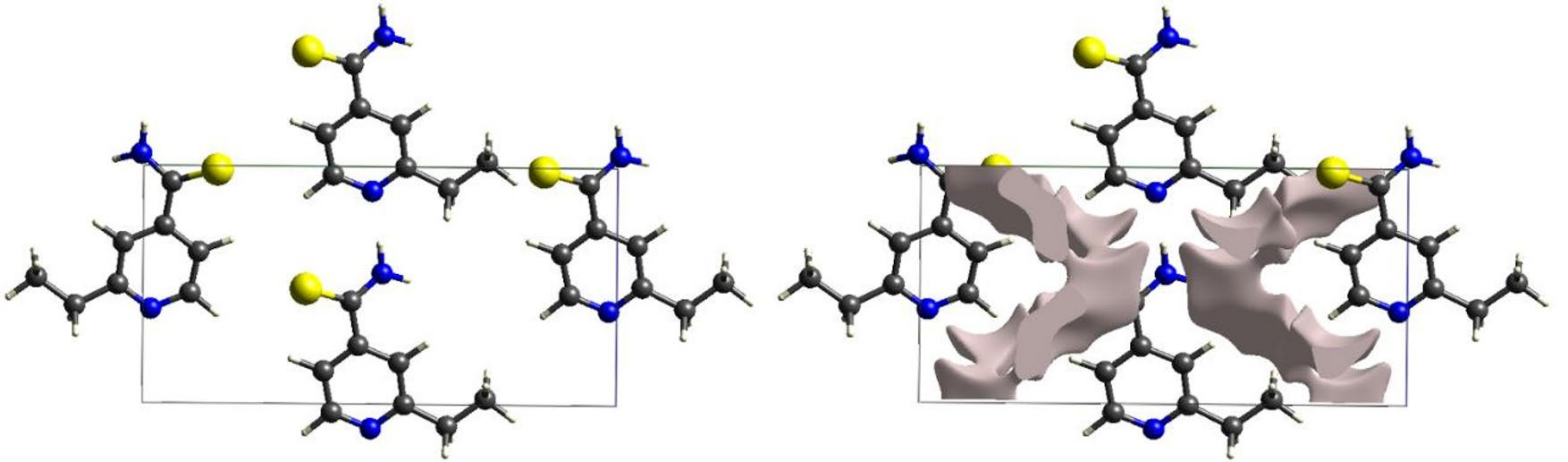
Crystal voids viewed along the *a*-axis in the unit cell through procrystal electron density isosurfaces (threshold = 0.002 a.u.) calculated using CrystalExplorer software. The plots were generated from CrystalExplorer software (version 21.5).

Another computational resource that provides valuable information about the unit cell is the crystal voids shown in Fig. 5. The void spaces are described through procrystal electron density isosurfaces that allow access to the physical data of the structure^36^. From this analysis, it was verified that the unit cell of the ETH crystal has a void volume of 43.47 Å^3^, corresponding to ≈ 5.3% of the total volume. According to the literature^27,50^, this percentage is classified as low, indicating that the chemical species in the structure may feature high lattice energy between their monomers. Furthermore, small impurities of low molecular volume or dopants of medium or small atomic radius can be introduced into this system to improve or achieve desirable properties. In addition, the places where the isosurfaces (occupancy area of 184.13 Å^2^) are not complementary closed represent the regions of the surface where the chemical groups of the molecule establish intermolecular contacts^52^. Moreover, it is significant to highlight that these data have not been previously reported in the literature for ETH.

### Energy frameworks analysis

To quantitatively decipher the anisotropic nature of intermolecular interactions that govern the crystal packing and stability of ETH, we employed energy framework analysis within CrystalExplorer. This method allows for the visualization and quantification of the direction-dependent interaction energies between the ETH molecules, decomposed into their fundamental components: Coulombic (*E*_Coul_), dispersion (*E*_disp_), electrostatic(*E*_ele_), and the total energy (*E*_total_) frameworks^49^, as depicted in Fig. S1a–d, respectively. The individual energy and total energy components for the calculated energy framework maps are given in Table S1.

The analysis reveals a striking dominance of dispersion forces throughout the crystal lattice, accounting for approximately 60% of the total stabilization energy. This observation is in good agreement with the Hirshfeld surface analysis (“Analyzes of Hirshfeld surfaces and their different mappings”), which identified H⋯H (49.3%) and H⋯S/S⋯H (22.1%) as the predominant contacts. The dispersion energy framework shows robust, relatively isotropic cylindrical tubes, indicating that these van der Waals interactions provide a foundational, omnidirectional cohesion to the structure.

In contrast, the Coulombic energy framework (Fig. S1a) exhibits significant anisotropy. Stronger and more directional interactions are observed along the crystallographic *a*-axis and within the *bc*-plane. These can be attributed to the more specific and polar interactions, such as the N–H⋯S and C–H⋯N hydrogen bonds identified earlier. The directionality of these electrostatic interactions can influence the preferred growth axes of the crystal.

The total energy framework (Fig. S1d) is the resultant of all components and provides the most critical insight for understanding bulk properties like solubility. It shows that the strongest overall stabilization propagates primarily along the *b*-axis. This suggests the formation of highly stable molecular chains linked via synergistic dispersion and electrostatic contacts, primarily involving the thiocarbonyl and pyridine groups. Consequently, crystal faces perpendicular to this direction, as the (010) family of planes, are expected to be the most stable and energetically costly to dissolve.

This directional bonding has direct implications in the crystal morphology and dissolution behavior. The anisotropic *E*_total_ framework predicts a plate-like or prismatic crystal habit, with the largest and morphologically most important faces being those parallel to the direction of strongest bonding (e.g., (010)), as these grow the slowest. These stable faces, characterized by their high lattice energy density, will present the greatest kinetic barrier to dissolution, directly contributing to ETH’s notoriously low solubility. Therefore, over the last few years, researchers have sought to synthesize new solid dispersions of ETH involving different coformers to overcome the low solubility of this drug.

This analysis correlates with the void calculation (“Analyzes of Hirshfeld surfaces and their different mappings”). The low void volume (5.3%) signifies dense, efficient packing, which is energetically stabilized by the strong and anisotropic interactions visualized in the energy framework. This combination of high lattice energy and directional stability creates a significant thermodynamic and kinetic barrier to solvation, reasoning the classification of ETH as a BCS Class II drug. Therefore, strategies to enhance solubility must target these specific, strong directional interactions. For example, cocrystallization with a coformer designed to disrupt the dominant *b*-axis stacking or surface functionalization of the (010) faces could provide a viable pathway to improved dissolution kinetics and bioavailability. Furthermore, the quantitative breakdown of intermolecular contacts and the anisotropic nature of the stabilization energy provide a blueprint for rational crystal engineering. Specifically, the strong interactions along the *b*-axis represent a key target for coformers aimed at disrupting the crystal lattice to enhance solubility.

### Thermal behavior

**Fig. 6:**
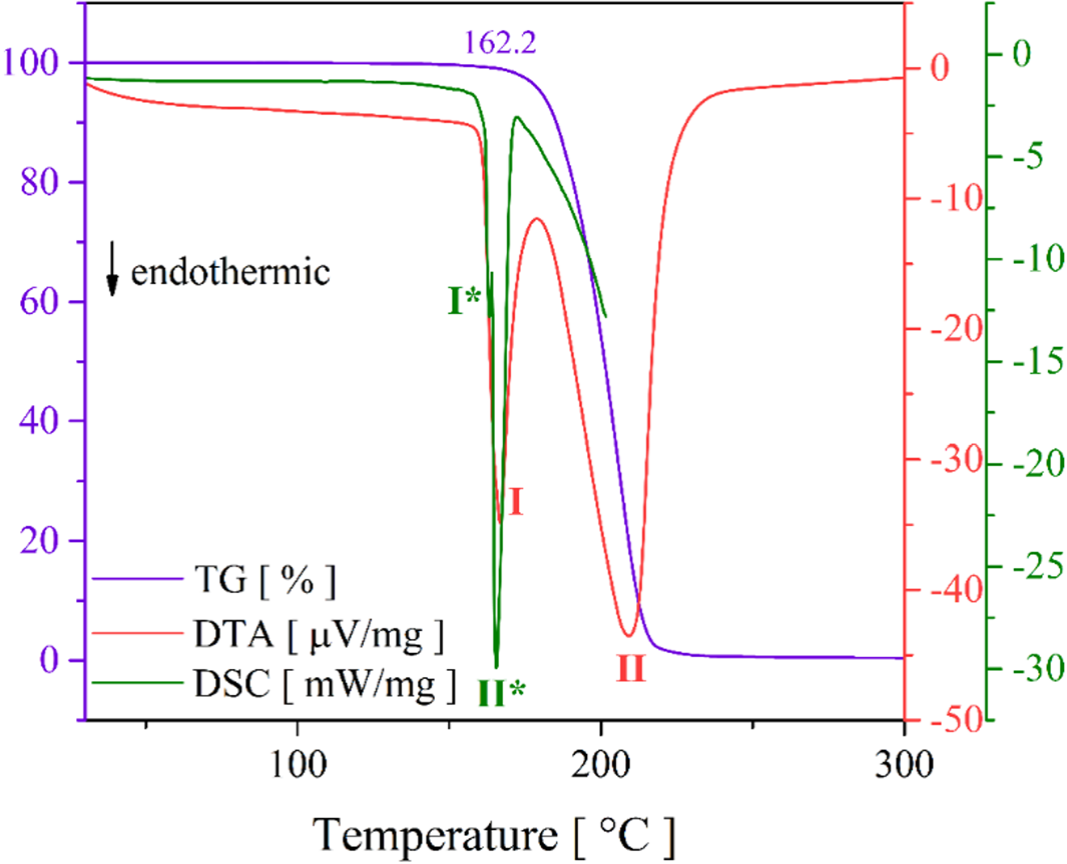
TG-DTA and DSC thermograms for the ETH powder crystal.

Figure 6 presents the TG-DTA and DSC measurements for the ETH powder crystal in the 30 to 300 °C range. The TG curve indicates no mass loss up to approximately ≈ 162.2 °C, indicating its thermal stability. Above this temperature, specifically between 162.6 and 172.5 °C, a sharp endothermic peak (I) is observed in the DTA curve, accompanied by a slight mass loss of about −7.1% (0.395 mg). This event corresponds to the ETH fusion process and likely involves the disruption of intermolecular interactions, such as hydrogen bonds, which stabilize the crystal lattice. The variation in heat may lead to the rupture of contacts like H⋯H, S⋯H/H⋯S, and N⋯H/H⋯N, thereby favoring the phase transition from solid to liquid. This same event is reflected in the DSC curve by two overlapping endothermic peaks at 163.2 (I*) and 165.7 (II*) °C, with enthalpy energy close to ≈ 10.8 kJ/mol. At higher temperatures, beyond 181.1 °C, the TG curve shows a marked decline with a mass loss of −91.2% (5.03 mg), related to the total decomposition of organic compounds. At around 230.0 °C, the absence of any mass percentage suggests that the drug is fully degraded, leaving no residual substances. Additionally, the broad endothermic peak (II) in the DTA range between 181.1 and 214.5 °C confirms this decomposition event.

### Geometric and electronic studies from the DFT calculations

**Fig. 7:**
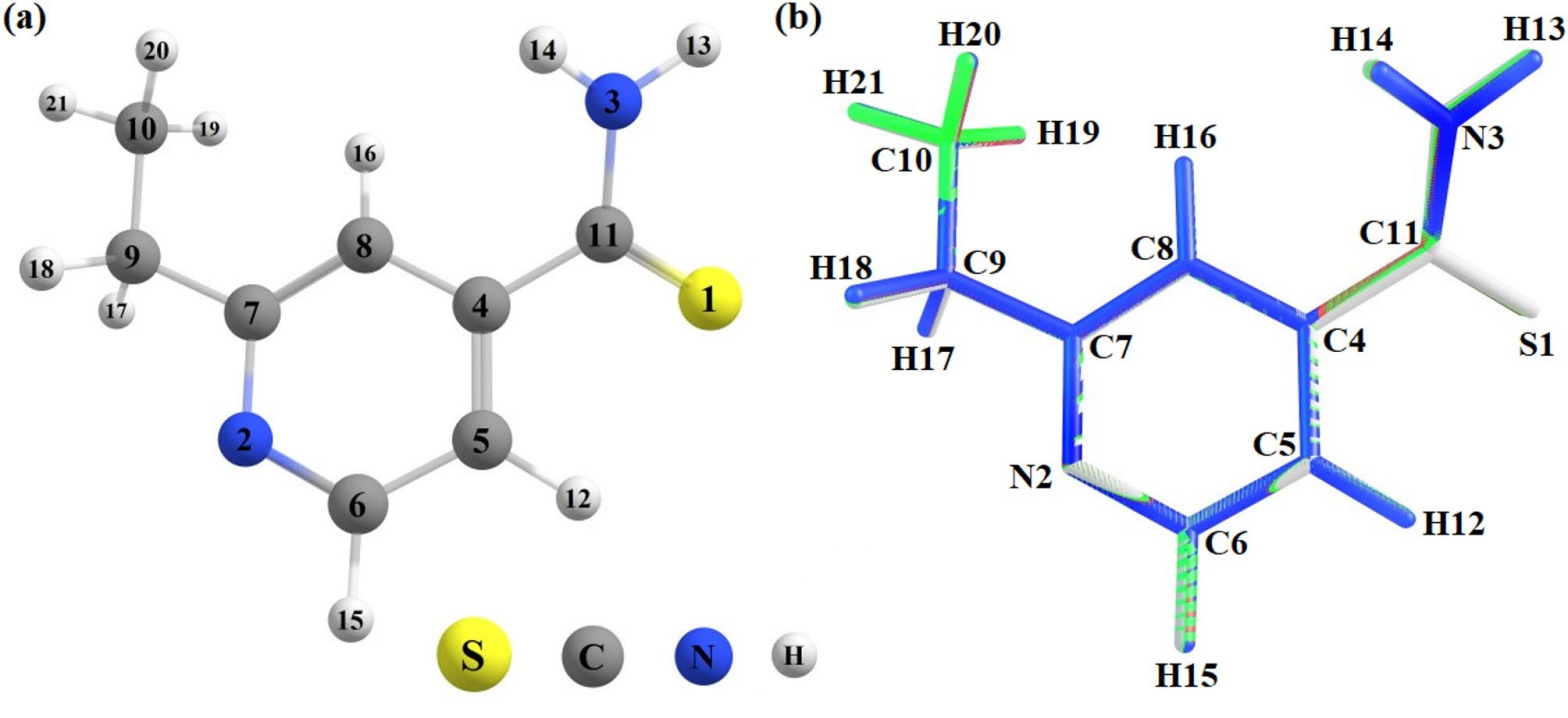
(**a**) Molecular structure of ETH optimized in the gas phase from the ωb97xd/6-311 + + g(d, p) method. (**b**) Overlay of the optimized ETH geometries in different solvents, demonstrating minimal conformational variation (RMSD < 0.03 Å between structures). The colors represent: chloroform (green), methanol (red), water (blue), and vacuum (gray). The plots were generated from Chemcraft software (version 1.8).

The ETH monomer geometry was optimized using the ωB97X-D functional and 6-311 + + G(d, p) basis set (Fig. 7a), followed by vibrational frequency analysis confirming the absence of imaginary modes (local minimum). Conformational searches around rotatable bonds (e.g., C9–C10 and C11–N3) confirmed the global minimum energy structure, with deviations < 1 kcal/mol from alternative conformers. Furthermore, computations were performed in some solvents, including chloroform, methanol, and water to assess the implicit solvation effect on the structure. Figure 7b shows the overlay of relaxed ETH monomer geometries in different solvents, highlighting minimal structural distortions. Although the overall backbone conformation remains stable, subtle variations in bond angles are evident, particularly in the vicinity of the S1 and N3 atoms under polar solvation.

The data were analyzed in terms of free solvation energy ((Δ*G*_*solv*_), revealing that ETH exhibits a higher affinity for polar solvents, such as water (Δ*G*_*solv*_ = − 8.96 kcal/mol) and methanol (Δ*G*_*solv*_ = − 8.67 kcal/mol), compared to the less polar chloroform (Δ*G*_*solv*_ = − 6.08 kcal/mol). The calculated values of enthalpy (∆*H*) and of the total electronic energy with zero-point vibrational energy (Δ*E*_ZPVE_) across solvents (water, methanol, chloroform) show minimal differences (≤ 0.01 kcal/mol), which are within the expected numerical noise of DFT methods due to convergence thresholds, integration grid precision, and solvation model approximations (supplementary material: Table S2). The solvation free energy provides a more reliable metric for solvent affinity.

Notably, energy differences < 0.5 kcal/mol (as ∆*H* variations of 0.01 kcal/mol between solvents) may not reflect physical trends. Such thresholds are consistent with reported methodological uncertainties by Zhan et al.^53^. Thus, here, solvent stability rankings should prioritize ∆*G*_*solv*_, which shows larger, statistically meaningful differences (supplementary material: Table S2).

In addition to the influence of solvents on thermodynamic parameters, this behavior on geometric properties in terms of bond lengths and bond angles was also analyzed. Table 1 presents these data combined with experimental values previously reported in the literature^33^. The uncertainties listed for bond lengths and angles determined by single-crystal X-ray diffraction (structure previously reported by Alleaume et al.^33^) are in good agreement with the calculated geometric parameters, which show minor deviations possibly attributable to the polycrystalline nature of the sample. Based on the estimated data, it can be seen that the ωb97xd/6-311 + + g(d, p) method provides data in good correlation with the experimental values for all the media used. Furthermore, the Root Mean Square Deviation (RMSD) analysis confirms this interpretation, in which only small deviations were detected, mainly for methanol, chloroform, and water.

The good agreement between theoretical and experimental data indicates that the ωb97xd/6-311 + + g(d, p) method accurately describes the molecular structure, regardless of the medium. Although minor variations in geometric parameters were observed across different solvents (Table 1), quantitative analysis indicates that these differences are minimal (RMSD < 0.02 Å for bond lengths and < 0.2° for bond angles). These variations are comparable to or smaller than the typical deviations associated with DFT methods, suggesting that solvent permittivity has a limited influence on the geometry of ETH. Specifically, the largest differences were found in the angles involving the thioamide group (S1–C11–N3), with a maximum variation of 1.6° across solvents. It is noteworthy that the implicit solvation model (IEFPCM) employed does not account for specific solute–solvent interactions, such as localized hydrogen bonding, which could induce more substantial conformational changes. Therefore, although calculations in solvent provide valuable insights into electronic properties, structural conclusions should consider these limitations.

**Table 1:** Bond lengths [Å] and angles [°] from the literature and results computed using Chemcraft software with functional ωb97xd/6-311 + + g(d, p) base set in different solvation media and under vacuum conditions. Standard uncertainties for bond lengths and angles are provided in parentheses. The RMSD values were calculated relative to the experimental structure.

Bonds	X-ray [Ă]	Chloroform		Methanol		Water		Vacuum	
		Calc. [Ă]	RMSD [Ă]	Calc. [Ă]	RMSD [Ă]	Calc. [Ă]	RMSD [Ă]	Calc. [Ă]	RMSD [Ă]
S1–C11	1.68(1)	1.66	0.01	1.67	0.01	1.67	0.01	1.65	0.02
C11–N3	1.33(8)	1.33	0.00	1.33	0.00	1.33	0.00	1.34	0.01
C11–C4	1.50(7)	1.49	0.00	1.50	0.00	1.49	0.00	1.49	0.00
C4–C5	1.40(1)	1.39	0.00	1.39	0.00	1.39	0.00	1.38	0.00
C5–C6	1.39(2)	1.39	0.00	1.39	0.00	1.39	0.00	1.39	0.00
C6–N2	1.34(5)	1.33	0.01	1.33	0.00	1.33	0.01	1.33	0.01
N2–C7	1.34(5)	1.34	0.00	1.34	0.00	1.34	0.00	1.33	0.00
C7–C8	1.40(1)	1.39	0.00	1.39	0.00	1.39	0.00	1.39	0.00
C8–C4	1.40(1)	1.39	0.00	1.39	0.00	1.39	0.00	1.39	0.00
C7–C9	1.51(1)	1.51	0.00	1.51	0.00	1.51	0.00	1.51	0.00
C9–C10	1.53(1)	1.52	0.00	1.52	0.00	1.52	0.00	1.52	0.00
Bonds	X-ray [°]	Calc. [°]	RMSD [°]	Calc. [°]	RMSD [°]	Calc. [°]	RMSD [°]	Calc. [°]	RMSD [°]
S1–C11–N3	121.4(4)	123.0	0.0	123.0	0.1	123.0	0.1	122.7	0.2
S1–C11–C4	121.3(1)	122.1	0.1	121.8	0.1	121.8	0.1	122.8	0.2
C4–C11–N3	117.2(2)	114.9	0.2	115.1	0.2	115.1	0.2	114.4	0.1
C8–C4–C5	117.9(8)	118.5	0.1	118.6	0.1	118.6	0.1	118.3	0.0
C4–C5–C6	118.6(7)	118.0	0.1	117.9	0.1	117.9	0.1	118.0	0.0
C7–N2–C6	118.3(1)	118.5	0.1	118.5	0.1	118.5	0.1	118.3	0.0
C9–C7–C8	123.5(6)	122.8	0.1	122.8	0.1	122.7	0.1	123.0	0.0
C10–C9–C7	116.8(1)	116.3	0.2	116.3	0.1	116.3	0.1	116.4	0.0
N2–C6–C5	123.6(1)	123.9	0.1	123.8	0.1	123.8	0.1	124.0	0.0
N2–C7–C8	121.4(8)	121.6	0.1	121.6	0.1	121.6	0.1	121.7	0.0
C4–C8–C7	119.9(4)	119.5	0.1	119.5	0.2	119.5	0.1	119.6	0.0
RMSD_Total_			0.2		0.2		0.2		0.1

To enhance the structural analysis and gain deeper insight into the molecular configuration and chemical surroundings of the ETH, a theoretical NMR investigation was performed. This study utilized geometry optimizations under solvation conditions. The simulated^1^H and^13^C NMR spectra are illustrated in Figures S2 and S3, with the corresponding chemical shift values (δ_Calc_), referenced to TMS, detailed in Tables S3 and S4. According to the data, solvent variation had minimal influence on the δ_Calc_ values for both hydrogen and carbon spectra. Notably, the most significant deviations were found among carbon atoms, particularly those located within the pyridine ring of ETH.

The electronic parameters derived from DFT calculations include HOMO and LUMO energies, electronic HOMO-LUMO gap, ionization potential (*IP*), electron affinity (*EA*), electronegativity (*χ*), chemical potential (*µ*), hardness (*η*), softness (*ς*), electrophilicity index (*ω*), and dipole moment (DM)^54^, as summarized in Table 2. While DFT virtual orbitals do not rigorously describe two-particle excitations, the ωB97X-D functional has been empirically validated for predicting frontier orbital energies and related reactivity indices in organic systems, as demonstrated in studies of other organic compounds^14,38^. These approximations provide qualitatively consistent trends for comparative analysis, though absolute values may require higher-level methods for quantitative accuracy.

Figure S4 illustrates the spatial distribution of HOMO and LUMO in different solvation media. The HOMO surfaces are located around the pyridine ring and the amine (NH_2_) and thiocarbonyl (C=S) functional groups with energies ranging between −8.20 eV (vacuum) and −8.49 eV (water). Similarly, the projections of the LUMO orbitals are also located in the same chemical groups of the ETH molecule, however, they do not involve all hydrogen atoms belonging to the unit. Furthermore, the energy values for the LUMO orbitals in vacuum, methanol, chloroform, and water were −0.37 eV, −0.40 eV, −0.39 eV, and −0.40 eV, respectively. These low values obtained for the LUMO orbital suggest that the molecular system is susceptible to receiving electrons at the sites where the surfaces are concentrated. Additionally, the difference between the HOMO and LUMO energies provides a chemical reactivity index, called electronic gap, which allows describing the electrical nature of a molecule, as well as its reactivity. High gap values (gap_Vacuum_= 7.84 eV; gap_Methanol_= 8.08 eV; gap_Chloroform_= 8.00 eV; gap_Water_= 8.09 eV) were obtained for ETH in the different conditions explored (Supplementary material: Fig. S4). Such results point to the dielectric character of the molecule and indicate that the system is electronically stable^55^. The solvation free energies correlate with ETH experimental solubility trends^4^, suggesting preferential stabilization in polar media. However, the high gap values and low softness indicate kinetic stability, which may limit dissolution rates, a key challenge for ETH formulations. Table 2 summarizes all these electronic descriptors and other global chemical reactivity parameters.

**Table 2:** Calculated electronic descriptors (HOMO, highest occupied molecular orbital; LUMO, lowest unoccupied molecular orbital; gap, HOMO-LUMO; *IP*, ionization potential; *EA*, electron affinity; *χ*, electronegativity; *µ*, chemical potential; *η*, Hardness; *ς*, Softness; *ω*, electrophilicity index; and DM, dipole Moment) for ETH in different media (energies in eV) from the ωb97xd/6-311 + + g(d, p) method.

Medium	Chemical reactivity descriptors [eV]										
	HOMO	LUMO	gap	IP	EA	χ	µ	η	ς *	ω	DM**
Vacuum	− 8.20	− 0.37	7.84	8.20	0.37	4.28	− 4.28	3.91	0.12	2.34	4.24
Methanol	− 8.48	− 0.40	8.08	8.48	0.40	4.44	− 4.44	4.04	0.12	2.44	6.61
Chloroform	− 8.39	− 0.39	8.00	8.39	0.39	4.39	− 4.39	4.00	0.12	2.41	5.98
Water	− 8.49	− 0.40	8.09	8.49	0.40	4.44	− 4.44	4.04	0.12	2.44	6.69

*Energy in eV^− 1^.

**Value in debye (*D*).

The HOMO localization over the pyridine ring and thiocarbonyl group (see Fig. S4) aligns with ETH bioactivation site, where cytochrome P450 oxidizes the thioamide to a reactive sulfonic acid intermediate^9^. The low LUMO energy (−0.40 eV) further suggests susceptibility to nucleophilic attack, consistent with its prodrug mechanism.

Using the HOMO and LUMO energies, other global chemical reactivity indices were calculated to measure the electronic properties of ETH in different solvation media, as shown in Table 2. *IP* corresponds to the energy required to remove an electron from the HOMO of the ETH molecule, calculated via Koopmans’ theorem within DFT^53^. This approximation assumes frozen orbitals during ionization, neglecting electronic relaxation effects. This process results in the formation of a positive ion due to the loss of an electron. The values calculated for this descriptor varied between 8.20 (vacuum) and 8.49 eV (water). Complementarily, *EA* indicates the amount of energy released from an isolated atom when receiving an electron, making it an ion. According to the results, it was found that a low energy (0.37–0.40 eV) is released for the ETH molecule when receiving an electron. Knowing these properties is extremely important since this drug is widely used in pharmacology and interacts with several enzymatic sites to activate its pharmacodynamic principles^56^. Note that the *IP* and *EA* values derived from HOMO/LUMO energies via Koopmans’ theorem provide a first-order estimate. While computationally efficient, this approach does not account for orbital relaxation or electron correlation effects upon ionization/electron attachment. However, the trends observed here remain valid for comparative analysis across solvents. Additionally, *χ* indicates the tendency of a molecule to attract electrons, which is why the values calculated for this index are all positive. Also, the negative values obtained for the *µ* parameter indicate the tendency of ETH to accept electrons under different solvation conditions^53,57^.

In addition to the electronic gap, which is related to the chemical reactivity of a molecule, the parameters *η* and *ς* further detail this behavior^58^. These descriptors are linked to polarizability, with species that are poorly polarizable being classified as hard, and those that are highly polarizable as soft. The calculated softness (0.12 eV^−1^) and hardness (3.91–4.04 eV) values for ETH fall within intermediate ranges for organic drug molecules^55,59^. These indices suggest moderate polarizability, consistent with ETH balanced electronic stability and reactivity profile. Another parameter directly associated with the application of compounds in biological activity is the *ω* index. This index measures the propency of the molecule to accept electrons from the donor species to the receptor^60^. Interestingly, under the influence of a solvent, ETH exhibits higher values when compared to vacuum. These data suggest a potential enhancement in solvation energy, which could influence its interaction with biomolecular targets in microorganisms. However, further studies with explicit solvent models are required to elucidate specific molecular interactions. The higher *ω* values in polar solvents suggest that ETH exhibits greater electrophilic reactivity in biological environments, where the polarity is similar to that of water. This trait is essential for its enzymatic activation, since ETH is a drug that requires metabolism by specific enzymes to become active^61^. Previous studies show that molecules with higher *ω* tend to interact more efficiently with electron-rich electronic sites, such as those found in target enzymes^56,62^. This result highlights how easy a system can interact with a biomolecular target in several microorganisms. It is important to highlight that trends in reactivity indices across solvents reflect bulk dielectric screening rather than explicit solvent coordination, as IEFPCM does not model localized interactions like hydrogen bonding.

The DM descriptor, in addition to being related to the distribution of electrons in a molecule, is considered a descriptor associated with electron mobility, predicting how easy a system interacts with another molecular unit^59^. Under the effect of solvation, ETH presented values of 6.69 *D*, 6.61 *D*, and 5.98 *D* in water, methanol, and chloroform, respectively, while under vacuum conditions this value was lower (4.24 *D*).

**Fig. 8:**
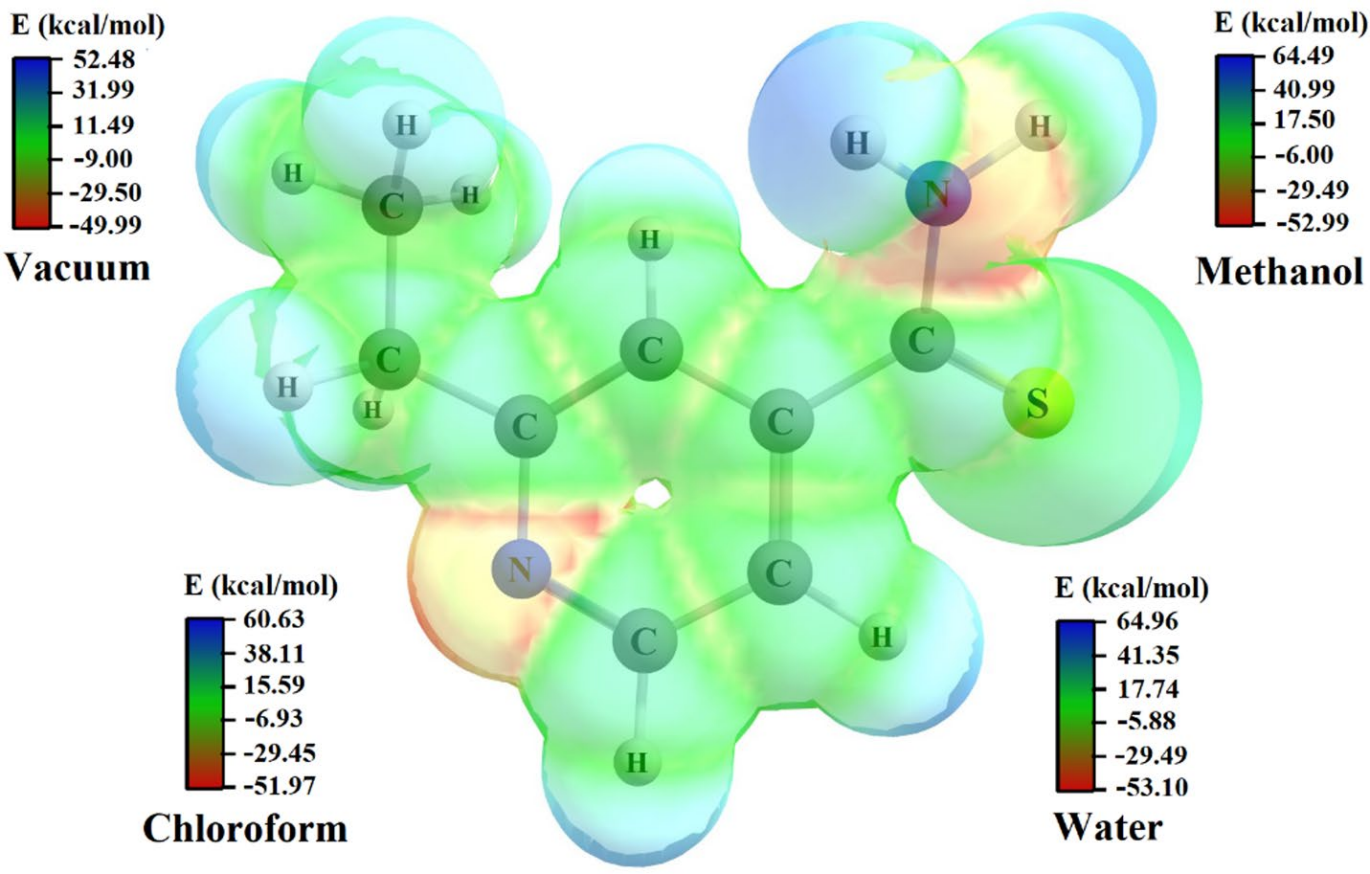
EPM of ETH in vacuum, methanol, chloroform, and water mapped on electron density surface using the DFT functional ωb97xd/6-311 + + g(d, p). The plots were generated from Chemcraft software (version 1.8). The spatial distribution of the electrostatic potential is qualitatively similar across solvents due to the limitations of the IEFPCM model, which does not induce specific solute-solvent reorganization.

An important computational tool for pharmacological inputs is the electrostatic potential map (EPM), which was calculated on the electron density isosurface (0.005 a.u.) using the Merz-Kollman scheme, with colors representing the local electrostatic potential (red: electron-rich/negative; blue: electron-deficient/positive). It is crucial to note that the implicit solvation model (IEFPCM) used here treats the solvent as a homogeneous dielectric continuum. While this approach effectively captures bulk polarization effects, leading to the changes in dipole moment and solvation energy reported in Table 2, it does not account for specific solute-solvent interactions, such as explicit hydrogen bonding. Consequently, the spatial distribution of the EPM is not significantly altered between different solvents. Therefore, the primary effect is a uniform scaling of the potential range, as observed in the color bars of Fig. 8. Thus, the following analysis focuses on the general features of the EPM relevant for solid-state interactions, while acknowledging that a detailed understanding of solvent-specific effects at crystal surfaces would require more advanced explicit solvation models.

Figure 8 presents the EPM with the estimated energy values for the different conditions. The orange-red regions correspond to the locations of the highest electron density, while the green and blue regions represent neutral electrostatic potential and low electrostatic potential zones, respectively. An equivalent pattern was observed for the four different media. The areas of negative electrostatic potential observed, particularly around atoms N, represent potential sites for hydrogen bonding and protonation. These interactions are highly relevant to ETH pharmacodynamic behavior, as they may facilitate binding to biological targets or influence solubility through intermolecular contacts. Given that ETH therapeutic efficacy is limited by its low solubility and bioavailability, identifying such reactive zones provides a molecular-level rationale for designing improved solid dispersions, such as salts, cocrystals, or coamorphous systems that exploit these interaction hotspots.

The quantitative energy ranges of the EPM differ (vacuum −2.49 kcal/mol; methanol −11.50 kcal/mol; chloroform −8.66 kcal/mol; water −11.86 kcal/mol), reflecting the uniform stabilization by the dielectric continuum. However, the overall spatial distribution remains qualitatively similar across media, confirming that solvation does not significantly redistribute the electron density profile. This is consistent with the limitations of the implicit model, which may underestimate asymmetric reorganization, particularly for strong, specific interactions like hydrogen bonds.

The dielectric environment’s role is more clearly captured by the calculated dipole moments, which increase with solvent polarity (from 4.24 D in vacuum to 6.69 D in water, Table 2), suggesting enhanced charge separation. This increased molecular polarity in polar solvents could, in principle, influence interactions with biological targets. However, as the EPM analysis underscores, specific binding interactions depend on explicit atomistic contacts not captured by the continuum model. Thus, while the dielectric environment modulates the electronic structure, definitive conclusions about pharmacological behavior require future studies with explicit solvent or protein-bound models.

### Vibrational properties combined with DFT calculations

**Fig. 9:**
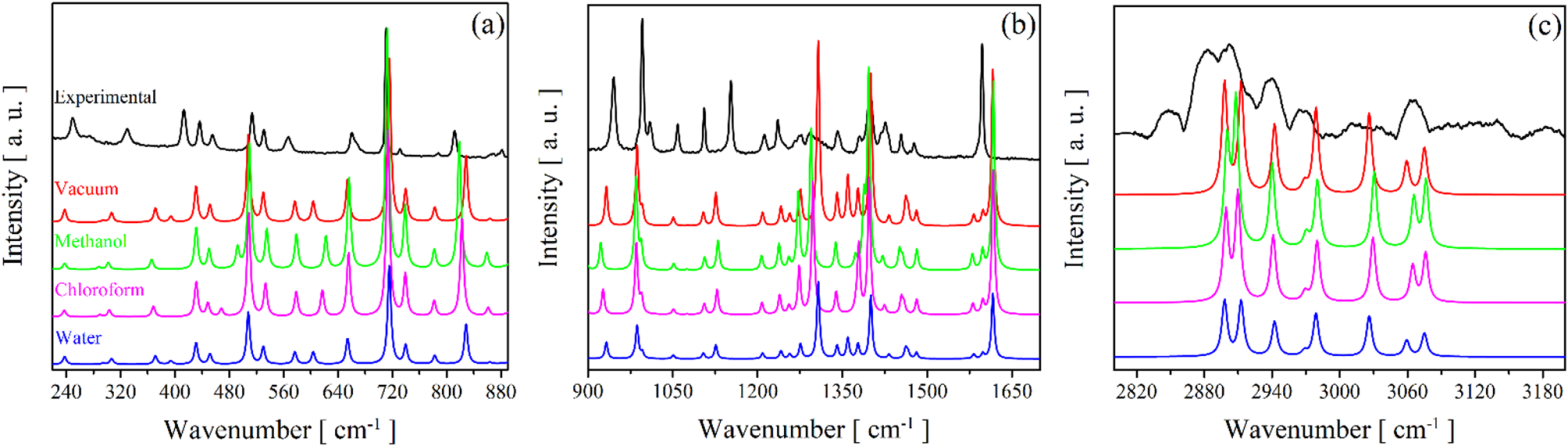
Comparison between calculated Raman spectra of ETH in vacuum, methanol, chloroform, and water using the DFT functional ωb97xd/6-311 + + g(d, p) in the spectral region: (**a**) 220–890 cm^− 1^, (**b**) 900–1700 cm^− 1^, and (**c**) 2800–3200 cm^− 1^.

**Fig. 10:**
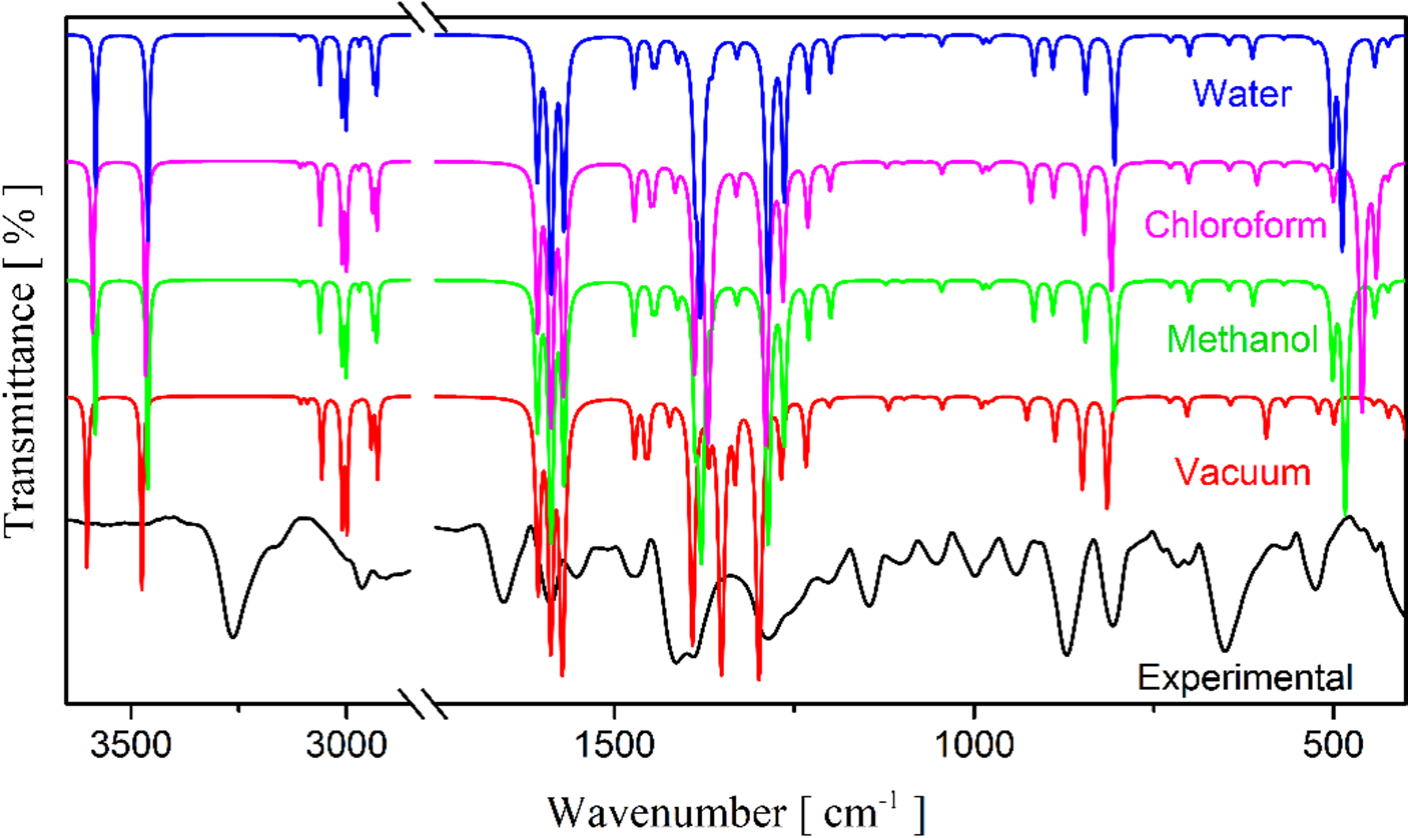
Comparison between calculated IR spectra of ETH in vacuum, methanol, chloroform, and water using the DFT functional ωb97xd/6-311 + + g(d, p).

Table 3 provides the positions of the experimental IR and Raman bands with their respective designations and wavenumbers calculated under solvation and vacuum conditions. The experimental and calculated Raman and IR spectra are presented in Figs. 9 and 10, respectively. In both spectra, in the high wavenumber region between 2900 and 3700 cm^− 1^, vibrational modes associated with stretching motions of the CH, CH_2_, and NH_2_ groups are observed. For example, the band recorded at 2963 cm^− 1^ in the IR spectrum is designated as symmetric stretching of the secondary methyl group. Similarly, this vibration was also detected in the Raman spectrum around 2964 cm^− 1^.

**Table 3:** Assignments of the main selected vibrational modes of ETH (IR_Exp_. – experimental IR band positions and R_exp_. Experimental Raman band positions). The calculated data were obtained under vacuum, chloroform, methanol, and water using the DFT functional ωb97xd/6-311 + + g(d, p).

ω_Cal_. [cm^− 1^]						Assignments
Vacuum	Chloroform	Methanol	Water	IR_Exp_.	*R*_Exp_.	
288	297	296	296	–	274	τ(ring) + wag(N3H13H14)
364	361	358	358	–	329	δ(ring) + wag(N3H13H14)
442	440	442	442	441	–	δ(ring) + wag(N3H13H14)
483	459	483	487	461	–	δ(ring) + wag(N3H13H14)
526	523	526	526	525	531	δ(ring) + δ(C10H19H20H21) + ρ(C9H17H18) + tw(N3H13H14)
569	568	569	568	564	567	δ(ring) + δ(C10H19H20H21) + ρ(C9H17H18)
645	643	645	646	651	660	ρ(C9H17H18) + δ(C4C11S1N3) + δ(ring)
700	701	700	701	701	–	δ(ring) + ν(C11S1)
726	726	726	722	716	710	δ(ring) + ρ(C9H17H18) + δ(C10H19H20H21)
768	768	768	769	737	731	ν(ring) + δ(C10H19H20H21) + ρ(C9H13H18)
805	808	805	804	806	811	ν(ring) + δ(C10H19H20H21) + δ(C9C7H18H17) + ρ(N3H14H15) + ν(C11S1)
889	895	889	890	871	881	tw(C9H17H18) + ν(C8H16) + δ(C10H19H20H21)
978	979	979	979	980	995	*br*(ring) + δ(C10H19H20H21) + wag(C9H17H18) + ρ(N3H14H15)
987	988	987	987	998	–	ν(ring) + δ(C10H19H20H21) + wag(C9H17H18) + ρ(N3H14H15)
1044	1044	1044	1045	1051	1058	ν(ring) + δ(C10H19H20H21) + δ(C9C7H18H17)
1099	1098	1099	1099	1100	1105	ν(ring) + δ(C10H19H20H21)
1122	1121	1122	1124	1146	1152	ρ(N3H14H15) + ν(ring)
1199	1200	1200	1199	1200	1212	wag(C9H17H18) + ν(ring) + ρ(N3H14H15)
1247	1247	1246	1247	1254	–	tw(C9H17H18) + δ(C10H19H20H21)
1286	1289	1286	1285	1285	1293	ρ(N3H14H15) + ν(C11N3) + ν(ring)
1387	1388	1379	1380	1389	1398	sc(N3H14H15) + ν(C11N3) + ν(ring)
1412	1415	1411	1412	1414	1426	sc(C9H17H18) + ν(ring)
1472	1471	1472	1472	1472	1469	sc(C10H21H20) + sc(C10H19H21)
1570	1570	1569	1569	1552		sc(N3H14H15) + ν(ring)
1588	1588	1587	1588	1588	1596	sc(N3H14H15) + ν(ring)
1607	1606	1607	1607	1653	–	ν(ring)
2930	2929	2930	2930	2907	2902	ν_s_(C10H20H21) + ν_s_(C10H219H21) + ν_s_(C10H20H29)
2970	2970	2970	2971	2963	2964	ν_s_(C9H17H18)
3108	3107	3108	3108	3161	–	ν(C6H15) + ν(C5H12)
3461	3465	3461	3460	3472	–	ν_s_(N3H14H15)
3583	3588	3582	3582	3608	–	ν_as_(N3H14H15)

*τ, torsion; ω, wagging; sc, scissoring; ν, stretching; ν_a_, anti-symmetric stretching; ν_s_, symmetric stretching; δ, bending; tw, twisting; wag, wagging; ρ, rocking. *br*, breathing.

**Scaled frequencies (0.957×) improve agreement with experimental data. Modes involving C = S (e.g., 701 cm^− 1^) show larger deviations due to crystal packing effects.

The Raman and IR bands recorded between 710 and 1700 cm^− 1^ are mainly related to movements of the pyridine ring with minor contributions from the NH_2_, CH_2_, CH_3_, and CN groups, with emphasis on the mode at 1146 cm^− 1^ in the Raman spectrum associated with the rocking vibration of NH_2_ and stretching of the pyridine ring. The same vibration was also seen in the Raman spectrum close to 1152 cm^− 1^. This mode corresponds to a vibration that, in the crystal, may overlap spatially with contacts between monomers; in our monomer level IEFPCM calculations, such intermolecular interactions are not explicitly modeled.

In the low wavenumber region of the IR spectrum (440–700 cm^− 1^), low transmittance bands—characteristic of almost all functional groups of the ETH molecule vibrating simultaneously – are observed. The IR absorption band located around 701 cm^− 1^ corresponds to a deformation of the pyridine ring and the symmetric C–S stretching. Furthermore, another mode involving the sulfur atom was recorded around 651 cm^− 1^ (ρ(CH_2_) + δ(CCSN) + δ(ring)). This vibration was also shown in the Raman spectrum at 660 cm^− 1^. These modes involving the S atom are presented in this range due to their molecular mass, being higher than the other constituents. Generally, atoms with high molar mass are recorded in the lower wavenumber region, as reported in the literature^63,64^. While monomer calculations capture most vibrational modes, discrepancies observed in the 500–1000 cm^− 1^ range are likely attributable to intermolecular interactions in the crystalline environment, particularly C=S∙∙∙H contacts identified in Hirshfeld surface analysis. These observations are consistent with crystal packing effects that are not fully captured by isolated monomer calculations. As noted in our methodological discussion, future studies employing periodic DFT calculations will provide more detailed insights into these solid-state vibrational perturbations.

Between 200 and 400 cm^− 1^ in the Raman spectrum, only deformation and torsion modes of chemical groups are recorded. Furthermore, the 200 cm^− 1^ modes not reported here are associated with lattice modes, i.e., vibrations corresponding to intermolecular interactions between chemical species. As in our calculations using only one monomeric unit, the assignment was directed to intramolecular modes.

The vibrational fingerprints not only characterize ETH but also guide its optimization in the production of solid dispersions. The identified modes serve as markers for monitoring polymorphic purity during formulation, while the functional groups inform under the design of salts/cocrystals or coamorphous to enhance bioavailability. These vibrational features directly influence ETH pharmacological properties: (*i*) C = S and NH_2_ stretching modes reflect hydrogen-bonding potential, which is critical for crystal packing and solubility modulation (*ii*) pyridine ring vibrations (e.g., 1588 cm^− 1^) correspond to molecular regions capable of interacting with the double-stranded DNA of pathogenic organisms, thereby contributing to antimicrobial activity; (*iii*) low-frequency torsional motions (< 300 cm^− 1^) suggest enhanced molecular flexibility, which can be strategically exploited in co-formulation approaches.

These assigned vibrational modes not only characterize the pure ETH but also serve as essential benchmarks for monitoring the formation and stability of future solid dispersions, such as cocrystals, salts or amorphous phases, ensuring the desired molecular interactions are achieved.

## Conclusions

Our integrated experimental-computational study provides fundamental insights into ETH solid-state properties and their direct implications for pharmaceutical development. The crystal structure analysis reveals a monoclinic system (C1*c*1($\:{C}_{s}^{4}$)-space group) stabilized by strong H⋯H (49.3%) and H⋯S/S⋯H (22.1%) interactions, which create a densely packed lattice with only 5.3% void space. Energy framework analysis quantitatively deciphered the anisotropic nature of these intermolecular interactions, revealing a striking dominance of dispersion forces (≈ 60% of the total stabilization energy) and directional Coulombic interactions along specific crystallographic axes. The strongest overall stabilization propagates along the *b*-axis, suggesting the formation of highly stable molecular chains. These structural features correlate with the compound’s high thermal stability (up to 162.2 °C) and help explain its well-documented solubility challenges as a BCS Class II drug.

The DFT calculations yield several pharmacologically relevant findings: the large HOMO-LUMO gap (7.83–8.09 eV) suggests low intrinsic reactivity but may contribute to slow dissolution kinetics, while the greater thermodynamic stability in polar solvents (Δ*G*_*solv*_ = −8.96 kcal/mol in water) aligns with experimental solubility trends. Notably, the HOMO localization on the thioamide group indicates this moiety’s dual role in both crystal cohesion and enzymatic activation, providing molecular-level insight into ETH prodrug mechanism. Furthermore, the increase in dipole moment with solvent polarity (from 4.24 D in vacuum to 6.69 D in water) suggests a modulation of the molecule’s electronic environment, which could influence its behavior in biological systems.

Although solvation studies have indicated greater thermodynamic stability in polar solvents, geometric and theoretical NMR analyses revealed that the molecular structure of ETH remains essentially unchanged across different media (variations < 0.5° in key bond angles; minimal influence on δ_Calc_ values). This reinforces that the solubility challenges of ETH are primarily associated with its strong intermolecular interactions in the solid state, rather than solvent-induced conformational changes.

Good agreement between experimental and calculated vibrational spectra establishes robust spectroscopic markers for pharmaceutical quality control, particularly the characteristic NH_2_ (3460 cm^− 1^) and C = S (700 cm^− 1^) vibrations. The comprehensive Hirshfeld surface and energy framework analyses quantitatively confirm the dominance of hydrogen bonding and van der Waals interactions that govern the crystal packing behavior, with dispersion providing a foundational, omnidirectional cohesion.

These findings suggest that conventional formulation approaches may be insufficient to overcome ETH strong crystal lattice. The demonstrated combination of high lattice energy and dense molecular packing necessitates innovative strategies such as coamorphous systems or targeted cocrystallization. Future research should incorporate explicit solvation models or periodic DFT simulations to better capture specific solvent-solute interactions, particularly at crystal surfaces, which are critical for understanding dissolution behavior and bioavailability.

These findings provide a clear molecular-level rationale for ETH poor solubility and directly suggest several actionable strategies for formulation improvement. The dominance of strong, directional intermolecular interactions, particularly the dispersion-driven packing along the b-axis and the N–H···S hydrogen bonds, creates a high-energy barrier to dissolution. Therefore, simply modifying crystal size or using standard surfactants may be insufficient. We propose that future efforts should focus on targeted solid-state engineering to disrupt these specific interactions. For instance: (*i*) cocrystallization—designing a cocrystal with a coformer containing complementary hydrogen bond acceptors (e.g., carboxylic acids) could compete with and disrupt the strong N–H···S and N–H···N motifs, potentially leading to a lattice with higher solubility; (*ii*) amorphization—the formation of a coamorphous system is a highly promising path. The vitreous state inherently lacks long-range order, effectively bypassing the energetic penalty of breaking the specific crystalline contacts identified here; (*iii*) surface functionalization—the energy framework analysis suggests that the (010) crystal face is particularly stable. Targeted adsorption of polymers or surfactants on this face could specifically reduce the dissolution barrier. The vibrational fingerprints and Hirshfeld surface analyses presented here will be crucial for characterizing the success of such strategies, serving as markers for polymorphic purity and interaction changes in new solid forms. By bridging fundamental solid-state properties with rational formulation design, this work provides a concrete roadmap for developing more bioavailable ETH-based therapies against MDR-TB.

## Supplementary Information

Below is the link to the electronic supplementary material.

Supplementary Material 1

## Data Availability

The initial structural data of ETH was retrieved from the Cambridge Structural Database (CSD), with reference code 1000256. The datasets generated and/or analysed during the current study are available in the Cambridge Crystallographic Data Centre (CCDC), https://www.ccdc.cam.ac.uk/structures/Search?cdcid=1000256&DatabaseToSearch=Published.
