# Supplementary material for: Experimental and computational analyses for elucidation of structural, electronic, thermal, and vibrational properties of ethionamide crystal
Source: Sci Rep. 2026 Jan 4;16:255. doi: 10.1038/s41598-025-29051-w (PMC12770466; doi:10.1038/s41598-025-29051-w)
Supplement: Supplementary file 1 — Supplementary Material 1 [file 41598_2025_29051_MOESM1_ESM.docx]

**Supplementary Information**

**Experimental and computational analyses for elucidation of structural, electronic, thermal, and vibrational properties of ethionamide crystal**

Raychimam D. S. Bezerra^1^, Ketelly E. da S. Alves^1^, Jailton R. Viana^1^, Luzeli M. da Silva^1^, Mateus R. Lage^1^, Rossano Lang^2^, Adenilson O. dos Santos^1^, Eliana B. Souto^3,^*, João G. de Oliveira Neto^1,^*

1. Center for Social Sciences, Health and Technology, Federal University of Maranhão - UFMA, 65900-410 Imperatriz, MA, Brazil
2. Institute of Science and Technology, Federal University of São Paulo-UNIFESP, 12231-280 São José Dos Campos, SP, Brazil
3. UCD School of Chemical and Bioprocess Engineering, University College Dublin, Belfield, Dublin 4, D04 V1W8, Ireland

***Corresponding authors:**

Eliana B. Souto, UCD School of Chemical and Bioprocess Engineering, University College Dublin, Belfield, Dublin 4 D04 V1W8, Ireland, [eliana.souto@ucd.ie](mailto:eliana.souto@ucd.ie)

João G. de Oliveira Neto, Center for Science of Imperatriz, Federal University of Maranhão – UFMA, 65900-410, Imperatriz, MA, Brazil; [joao.gon@ufma.br](mailto:joao.gon@ufma.br)

**Table S1.** Calculated interaction energies for the ETH molecules in kJ/mol, using electron density B3LYP/6–311G(d,p).

|  | N | Symop | R | *E*_ele_ | *E*_pol_ | *E*_dis_ | *E*_rep_ |
| --- | --- | --- | --- | --- | --- | --- | --- |
|  | 2 | x, y, z | 33.86 | - | - | - | - |
|  | 1 | x+1/2, y+1/2, z+1/2 | 25.09 | -0.1 | - | - | - |
|  | 1 | x+1/2, -y, z | 21.55 | 0.1 | - | - | - |
|  | 2 | x, -y+1/2, z+1/2 | 25.82 | 0.1 | - | - | - |
|  | 1 | x, -y+1/2, z+1/2 | 30.89 | - | - | - | - |
|  | 2 | x, y, z | 16.93 | -0.2 | - | - | - |
|  | 1 | x+1/2, -y, z | 31.32 | 0.1 | - | - | - |
|  | 2 | x, y, z | 21.79 | 0.0 | - | - | - |
|  | 1 | x+1/2, -y, z | 33.92 | - | - | - | - |
|  | 2 | x, y, z | 30.96 | -0.1 | - | - | - |
|  | 1 | x+1/2, -y, z | 17.18 | 0.4 | - | - | - |
|  | 1 | x+1/2, y+1/2, z+1/2 | 22.87 | -0.2 | - | - | - |
|  | 2 | x, -y+1/2, z+1/2 | 23.26 | 0.2 | - | - | -0.1 |
|  | 2 | x, y, z | 14.96 | -0.4 | - | - | -0.1 |
|  | 1 | x+1/2, -y, z | 30.61 | 0.1 | - | - | - |
|  | 1 | x+1/2, -y, z | 15.85 | 0.6 | - | -0.1 | - |
|  | 1 | x+1/2, y+1/2, z+1/2 | 23.28 | -0.1 | - | - | - |
|  | 2 | x, y, z | 16.93 | -0.2 | - | - | - |
|  | 1 | x+1/2, -y, z | 31.92 | 0.1 | - | - | - |
|  | 2 | x, y, z | 30.96 | -0.1 | - | - | - |
|  | 1 | x+1/2, -y, z | 18.24 | 0.2 | - | - | - |
|  | 1 | x+1/2, y+1/2, z+1/2 | 26.19 | -0.1 | - | - | - |
|  | 2 | x, y, z | 21.79 | - | - | - | - |
|  | 1 | x+1/2, -y, -z | 35.01 | - | - | - | - |
|  | 2 | x, y, z | 33.86 | - | - | - | - |
|  | 1 | x+1/2, -y, z | 23.24 | - | - | - | - |
|  | 1 | x+1/2, y+1/2, z+1/2 | 13.48 | -0.2 | - | -0.1 | - |
|  | 1 | x+1/2, -y, z | 15.05 | -0.6 | - | -0.1 | - |
|  | 2 | x, -y+1/2, z+1/2 | 14.30 | - | - | -0.1 | - |
|  | 0 | x+1/2, y+1/2, z+1/2 | 20.35 | - | - | - | - |
|  | 1 | x, -y+1/2, z+1/2 | 21.35 | -0.1 | - | - | - |
|  | 2 | x, y, z | 7.92 | -52.2 | -13.1 | -15.5 | 68.2 |
|  | 2 | x, y, z | 15.85 | 0.2 | - | - | - |
|  | 1 | x+1/2, -y, z | 7.55 | -8.8 | -2.1 | -10.6 | 7.1 |
|  | 1 | x+1/2, y+1/2, z+1/2 | 8.66 | -2.7 | -0.3 | -2.0 | 0.1 |
|  | 2 | x, -y+1/2, z+1/2 | 8.89 | 0.7 | -1.3 | -13.4 | 9.9 |
|  | 1 | x+1/2, -y, z | 3.61 | -43.8 | -7.3 | -57.3 | 86.7 |
|  | 1 | x+1/2, y+1/2, z+1/2 | 9.69 | -4.8 | -0.8 | -5.3 | 6.5 |
|  | 1 | x+1/2, -y, z | 9.73 | -1.4 | -0.3 | -1.5 | 0.2 |
|  | 1 | x+1/2, y+1/2, z+1/2 | 15.44 | 0.3 | - | - | -0.1 |
|  | 1 | x+1/2, -y, z | 17.37 | -0.2 | - | - | - |
|  | 1 | x+1/2, y+1/2, z+1/2 | 13.48 | 0.2 | - | -0.2 | - |
|  | 1 | x+1/2, -y, z | 20.88 | 0.1 | - | - | - |
|  | 2 | x+1/2, -y+1/2, z+1/2 | 13.80 | 0.1 | - | -0.1 | - |
|  | 0 | x+1/2, y+1/2, z+1/2 | 20.35 | 0.2 | - | - | - |
|  | 1 | x+1/2, -y+1/2, z+1/2 | 21.01 | - | - | - | - |
|  | 1 | x+1/2, -y, z | 16.33 | 0.3 | - | - | - |
|  | 1 | x+1/2, y+1/2, z+1/2 | 8.66 | -1.4 | -0.4 | -5.7 | 2.8 |
|  | 2 | x, -y+1/2, z+1/2 | 8.05 | -5.0 | -1.2 | -8.3 | 15.0 |
|  | 1 | x+1/2, -y, z | 14.93 | 0.7 | - | - | - |
|  | 1 | x+1/2, y+1/2, z+1/2 | 9.69 | 0.3 | -0.1 | -0.8 | - |
|  | 1 | x+1/2, -y, z | 17.45 | 0.2 | - | - | - |
|  | 1 | x+1/2, y+1/2, z+1/2 | 15.44 | - | - | - | - |
|  | 1 | x+1/2, -y, z | 22.62 | 0.1 | - | - | - |
|  | 1 | x+1/2, y+1/2, z+1/2 | 25.09 | -0.1 | - | - | - |
|  | 1 | x+1/2, -y, z | 33.07 | - | - | - | - |
|  | 2 | x+1/2, -y+1/2, z+1/2 | 24.98 | - | - | - | - |
|  | 0 | x+1/2, y+1/2, z+1/2 | 29.36 | 0.1 | - | - | - |
|  | 1 | x, -y+1/2, z+1/2 | 29.58 | - | - | - | - |
|  | 1 | x+1/2, -y, z | 30.40 | - | - | - | - |
|  | 1 | x+1/2, y+1/2, z+1/2 | 22.87 | 0.1 | - | - | - |
|  | 2 | x, -y+1/2, z+1/2 | 22.33 | -0.1 | - | - | - |
|  | 1 | x+1/2, -y, z | 29.67 | 0.2 | - | - | - |
|  | 1 | x+1/2, y+1/2, z+1/2 | 23.28 | 0.1 | - | - | - |
|  | 1 | x+1/2, -y, z | 31.02 | 0.1 | - | - | - |
|  | 1 | x+1/2, y+1/2, z+1/2 | 26.19 | - | - | - | - |
|  | 1 | x+1/2, -y, z | 34.19 | - | - | - | - |
|  | 1 | x+1/2, y+1/2, z+1/2 | 39.06 | - | - | - | - |
|  | 0 | x+1/2, -y, z | 46.89 | - | - | - | - |
|  | 2 | x, -y+1/2, z+1/2 | 38.80 | - | - | - | - |
|  | 0 | x+1/2, y+1/2, z+1/2 | 41.92 | - | - | - | - |
|  | 1 | x, -y+1/2, z+1/2 | 41.91 | - | - | - | - |
|  | 0 | x, y, z | 45.59 | - | - | - | - |
|  | 0 | x, y, z | 47.61 | - | - | - | - |
|  | 0 | x+1/2, -y, z | 45.05 | - | - | - | - |
|  | 1 | x+1/2, y+1/2, z+1/2 | 37.66 | - | - | - | - |
|  | 2 | x+1/2, -y, z | 37.15 | - | - | - | - |
|  | 0 | x, y, z | 44.89 | - | - | - | - |
|  | 0 | x+1/2, -y, z | 44.56 | - | - | - | - |
|  | 1 | x+1/2, y+1/2, z+1/2 | 37.91 | - | - | - | - |
|  | 0 | x, y, z | 45.59 | - | - | - | - |
|  | 0 | x+1/2, -y, z | 45.47 | - | - | - | - |
|  | 1 | x+1/2, y+1/2, z+1/2 | 39.77 | - | - | - | - |
|  | 0 | x, y, z | 47.61 | - | - | - | - |
|  | 0 | x+1/2, -y, z | 47.69 | - | - | - | - |
|  | 1 | x+1/2, y+1/2, z+1/2 | 43.03 | - | - | - | - |
|  | 1 | x+1/2, y+1/2, z+1/2 | 30.92 | - | - | - | - |
|  | 1 | x+1/2, y+1/2, z+1/2 | 22.54 | - | - | - | - |
|  | 1 | x+1/2, y+1/2, z+1/2 | 22.54 | 0.1 | - | - | - |
|  | 1 | x+1/2, y+1/2, z+1/2 | 30.92 | - | - | - | - |
|  | 1 | x+1/2, y+1/2, z+1/2 | 39.05 | - | - | - | - |
|  | 2 | x, -y+1/2, z+1/2 | 38.09 | - | - | - | - |
|  | 2 | x, -y+1/2, z+1/2 | 39.70 | - | - | - | - |
|  | 1 | x+1/2, y+1/2, z+1/2 | 37.66 | - | - | - | - |
|  | 1 | x+1/2, y+1/2, z+1/2 | 37.91 | - | - | - | - |
|  | 1 | x+1/2, y+1/2, z+1/2 | 39.77 | - | - | - | - |
|  | 1 | x, -y+1/2, z+1/2 | 42.75 | - | - | - | - |
|  | 1 | x+1/2, y+1/2, z+1/2 | 43.03 | - | - | - | - |

N- number of molecules; Symop- symmetry operation; R- average distance between molecules; *E*_ele_- electronic energy; *E*_pol_- polarization energy; *E*_dis_- dispersion energy; *E*_rep_- repulsion energy .

**Table S2.** Thermodynamic parameters calculated for ETH in different solvation media using the ωB97XD/6-311++G(d,p) method.

| Parameters | Energy [ kcal/mol ] | | |
| --- | --- | --- | --- |
|  | Water | Methanol | Chloroform |
| Δ*G_solv_* | –8.96 | –8.67 | –6.08 |
| ∆*H* | –818.38 | –818.38 | –818.37 |
| ∆*E*_ZPVE_ | –818.39 | –818.39 | –818.38 |

Energies with differences < 0.5 kcal/mol are considered indistinguishable within DFT error margins (see Section 3.4).

**Table S3.** ^13^C NMR chemical shifts calculated using the ωB97XD/6-311++G(d,p) method from TMS reference compound in ppm.

| Atoms | ωB97X-D/6–311++G(d,p) | | | |
| --- | --- | --- | --- | --- |
|  | Vacuum | Methanol | Chloroform | Water |
| C4 | 155.23 | 157.30 | 156.62 | 157.42 |
| C5 | 126.85 | 126.77 | 126.84 | 126.75 |
| C6 | 157.70 | 158.49 | 158.32 | 158.50 |
| C7 | 171.65 | 173.85 | 173.24 | 173.92 |
| C8 | 116.10 | 119.50 | 118.35 | 119.65 |
| C9 | 33.57 | 34.58 | 34.23 | 34.62 |
| C10 | 12.03 | 12.09 | 12.05 | 12.09 |
| C11 | 216.02 | 218.99 | 218.34 | 219.07 |

δ_Calc_= σ_TMS_ - σ.

**Table S4.** ^1^H NMR chemical shifts calculated using the ωB97XD/6-311++G(d,p) method from TMS reference compound in ppm.

| Atoms | ωB97X-D/6–311++G(d,p) | | | |
| --- | --- | --- | --- | --- |
|  | Vacuum | Methanol | Chloroform | Water |
| H12 | 7.90 | 7.85 | 7.88 | 7.84 |
| H13 | 6.87 | 7.54 | 7.36 | 7.56 |
| H14 | 6.20 | 7.02 | 6.77 | 7.05 |
| H15 | 8.92 | 8.96 | 8.96 | 8.96 |
| H16 | 7.25 | 7.54 | 7.42 | 7.56 |
| H17 | 3.10 | 3.06 | 3.08 | 3.06 |
| H18 | 3.10 | 3.06 | 3.08 | 3.06 |
| H19 | 1.32 | 1.36 | 1.35 | 1.36 |
| H20 | 1.21 | 1.36 | 1.30 | 1.36 |
| H21 | 1.42 | 1.43 | 1.44 | 1.43 |

δ_Calc_**=** σ_TMS_ - σ.


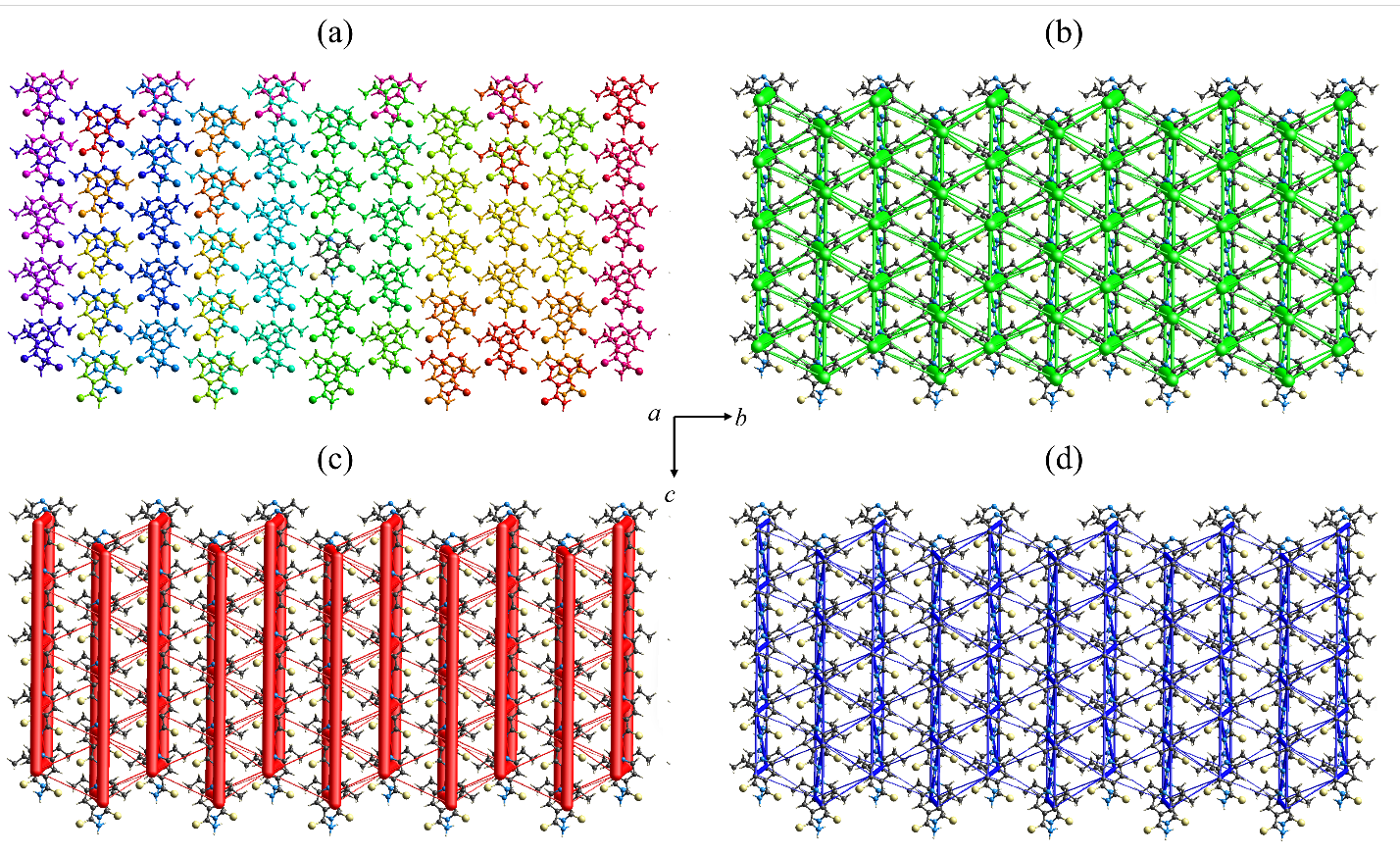


**Fig. S1.** Energy framework for ETH molecules mapped as a function of (a) Coulomb energy, (b) dispersion energy, (c) electrostatic energy, and (d) total energy.


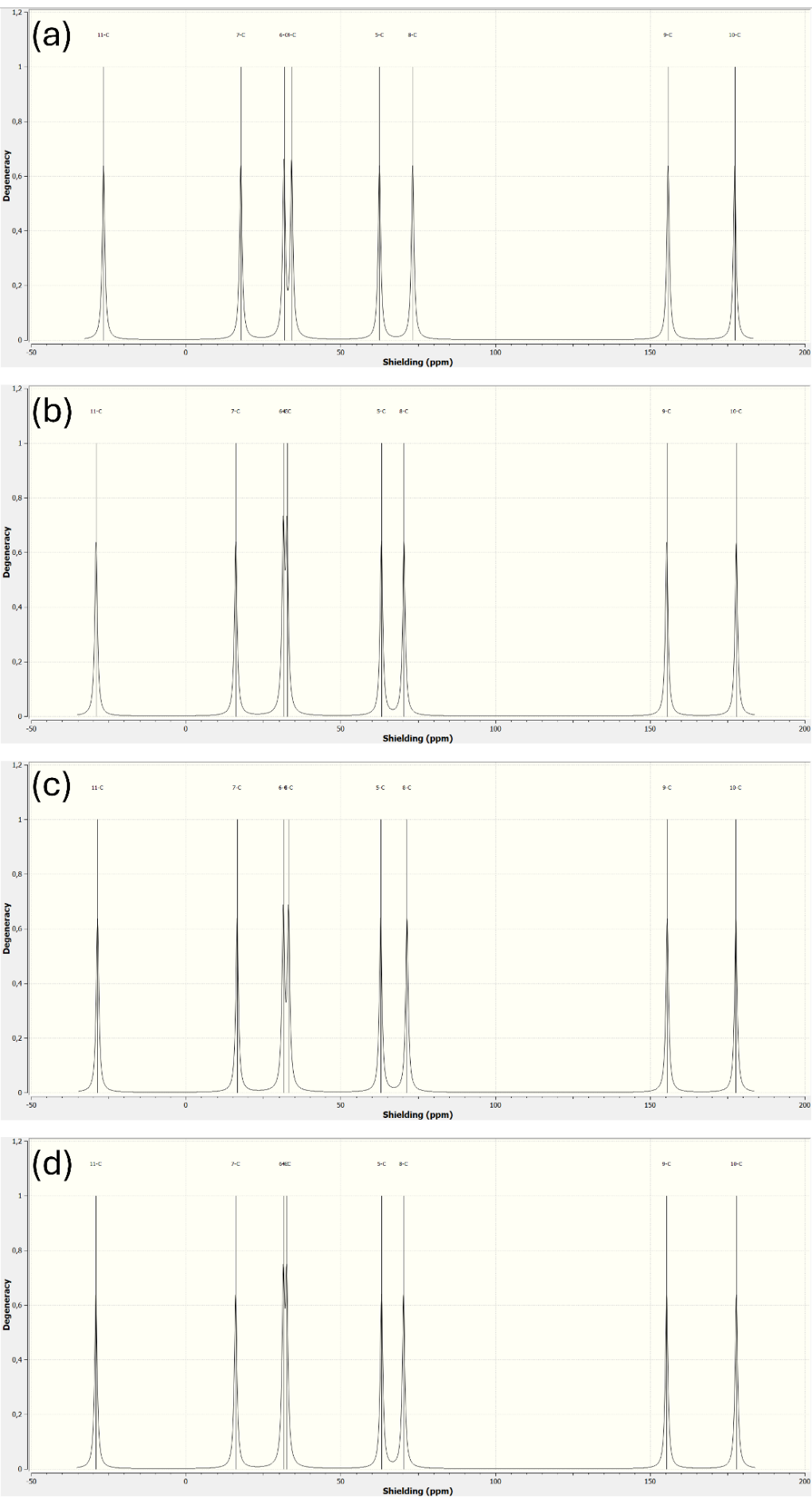


**Fig S2.** Calculated-^13^C RMN spectra for th ETH using the ωb97xd/6-311++g(d,p) method in conditions of (a) vacuum, (b) methanol (c) chloroform, and (d) water.


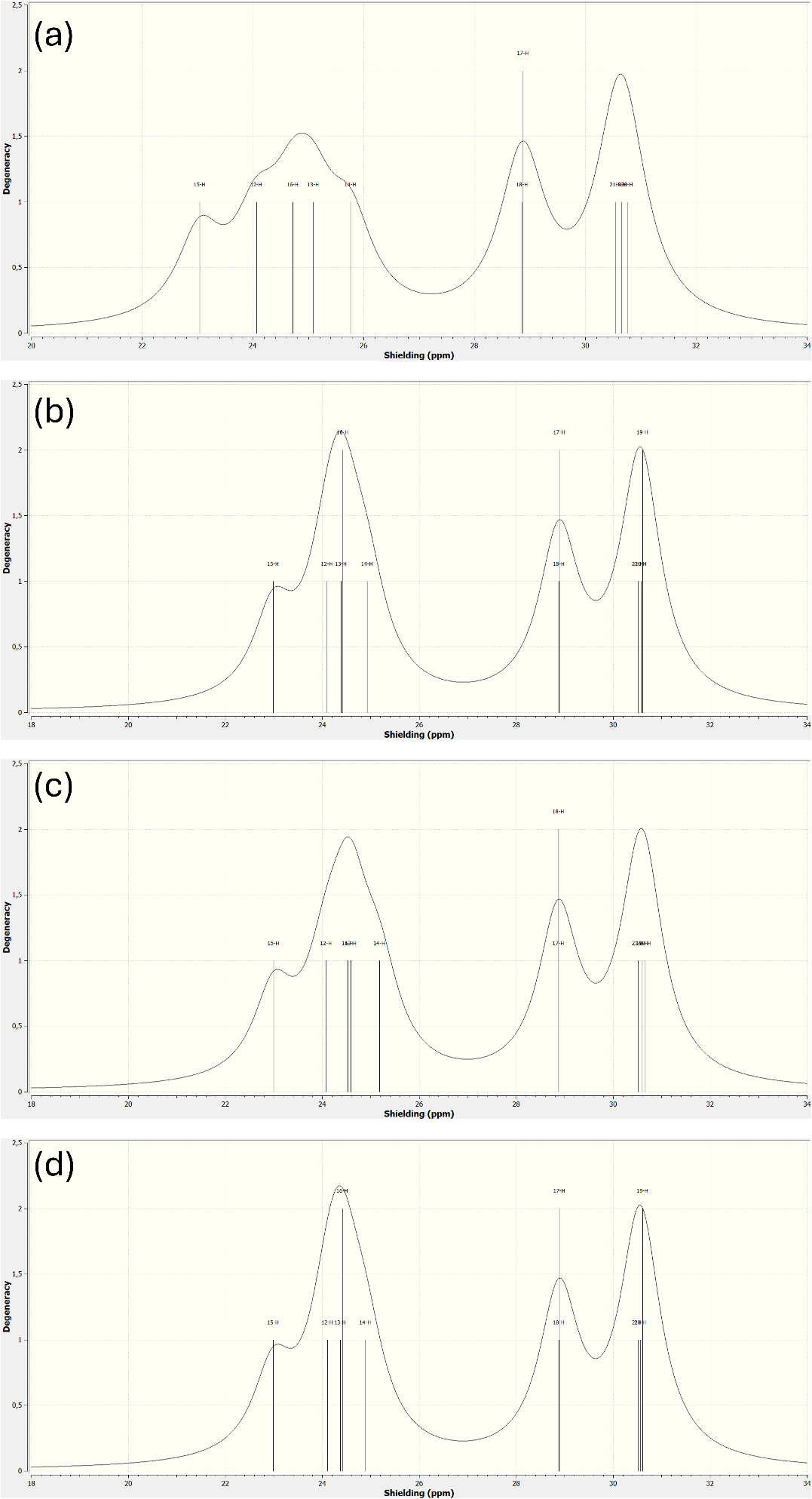


**Fig S3.** Calculated-^1^H RMN spectra for th ETH using the ωb97xd/6-311++g(d,p) method in conditions of (a) vacuum, (b) methanol (c) chloroform, and (d) water.


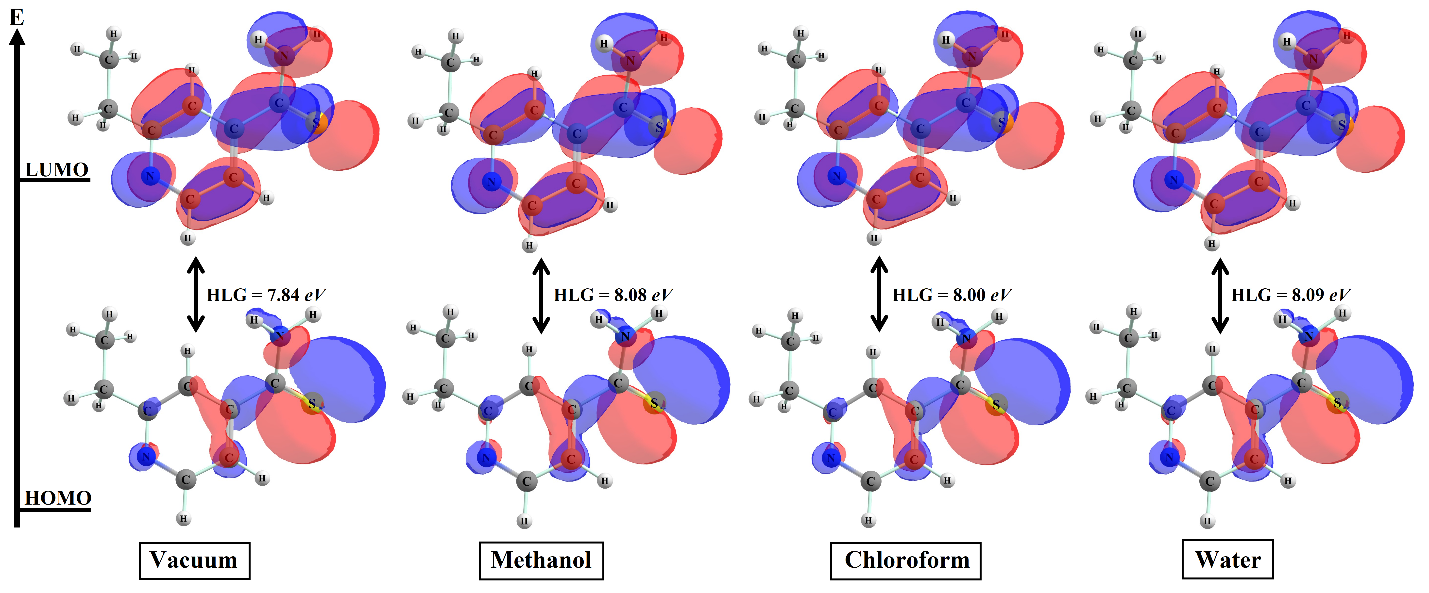


**Fig. S4.** HOMO and LUMO surface and HOMO-LUMO gap (HLG) plots for ETH calculated from the DFT functional ωb97xd/6-311++g(d,p) in vacuum, methanol, chloroform, and water. The plots were generated from Chemcraft software (version 1.8).
